# Black pepper (Piper nigrum L.) fruit extract ameliorates erectile dysfunction in alloxan-induced diabetic rats

**DOI:** 10.12688/f1000research.172637.1

**Published:** 2026-01-09

**Authors:** Exsa Hadibrata, Sutyarso Sutyarso, Hendri Busman, Syazili Mustofa, Wawan Abdullah Setiawan, Ratna Dewi Puspita Sari, Nuning Nurcahyani

**Affiliations:** 1 Doctoral Program, Universitas Lampung Fakultas Matematika dan Ilmu Pengetahuan Alam, Bandar Lampung, Lampung, Indonesia; 2 Department of Surgery, Universitas Lampung Fakultas Kedokteran, Bandar Lampung, Lampung, Indonesia; 3 Department of Biology, Universitas Lampung Fakultas Matematika dan Ilmu Pengetahuan Alam, Bandar Lampung, Lampung, Indonesia; 4 Department of Biochemistry and Biomolecular, Universitas Lampung Fakultas Kedokteran, Bandar Lampung, Lampung, Indonesia; 5 Department of Obstetric and Gynecology, Universitas Lampung Fakultas Kedokteran, Bandar Lampung, Lampung, Indonesia

**Keywords:** Diabetes Mellitus, Hyperglycemia, Black Pepper, Piper Nigrum, Erectile Function, Libido

## Abstract

**Background:**

Diabetes mellitus (DM) is a chronic metabolic disorder frequently associated with male sexual and reproductive dysfunction, including erectile dysfunction (ED), reduced libido, and impaired spermatogenesis. Black pepper ( *Piper nigrum* L.) fruit extract has been reported to possess antidiabetic and reproductive benefits, yet its effect on DM-related ED remains underexplored.

**Objective:**

To evaluate the effects of *Piper nigrum* fruit extract on erectile function, libido, sperm parameters, and testicular histology in alloxan-induced diabetic male rats.

**Methods:**

Thirty male Sprague Dawley rats were divided into five groups: normal control, diabetic control, diabetic rats treated with black pepper extract (122.5 or 245 mg/kg BW), and diabetic rats treated with sildenafil citrate. Erectile function was assessed via penile reflexes, libido by mating behavior, sperm quality by concentration, motility, and morphology, and testicular histology by Leydig cell and spermatogonia counts. Data were analyzed using ANOVA with significance at p<0.05.

**Results:**

Alloxan-induced diabetic rats showed significant impairment in erectile function, libido, sperm quality, and testicular histology (p<0.001 vs control). Black pepper extract at 122.5 mg/kg BW significantly improved total penile reflexes compared with diabetic controls (9.33±1.03 vs 6.00±1.26, p=0.02). Libido parameters including courtship latency (5.50±0.55 vs 21.00±9.47, p=0.013), mount latency (19.00±10.81 vs 37.17±6.31, p=0.009), and mount frequency (18.05±5.99 vs 7.17±1.83, p=0.002) were significantly improved. Sperm analysis revealed increases in sperm concentration (19.2±6.7 vs 12.6±1.3, though not significant, p=0.877), motility (31.8±23 vs 27±30, p=0.697), and normal morphology (40.9±7.8 vs 35±10.8, p=0.04). Testicular histology showed restoration of Leydig cell count (59.33±4.0 vs 30.50±3.86, p=0.035) and spermatogonia number (319.4±64.59 vs 491±37.0, p<0.001). The 245 mg/kg BW dose primarily improved sperm concentration (62.95±29.4 vs 12.6±1.3, p=0.01) and motility (36.6±23 vs 27±30, p=0.436). Sildenafil citrate significantly enhanced most parameters compared with diabetic controls (p<0.05).

**Conclusion:**

*Piper nigrum* fruit extract ameliorates sexual dysfunction and reproductive impairment in alloxan-induced diabetic rats, particularly at 122.5 mg/kg BW, with significant improvements in erectile function, libido (p<0.05), sperm quality (p≤0.04), and testicular histology (p=0.035). These findings suggest its potential as a natural therapeutic agent for DM-related male reproductive dysfunction.

## Introduction

**Figure 1.**
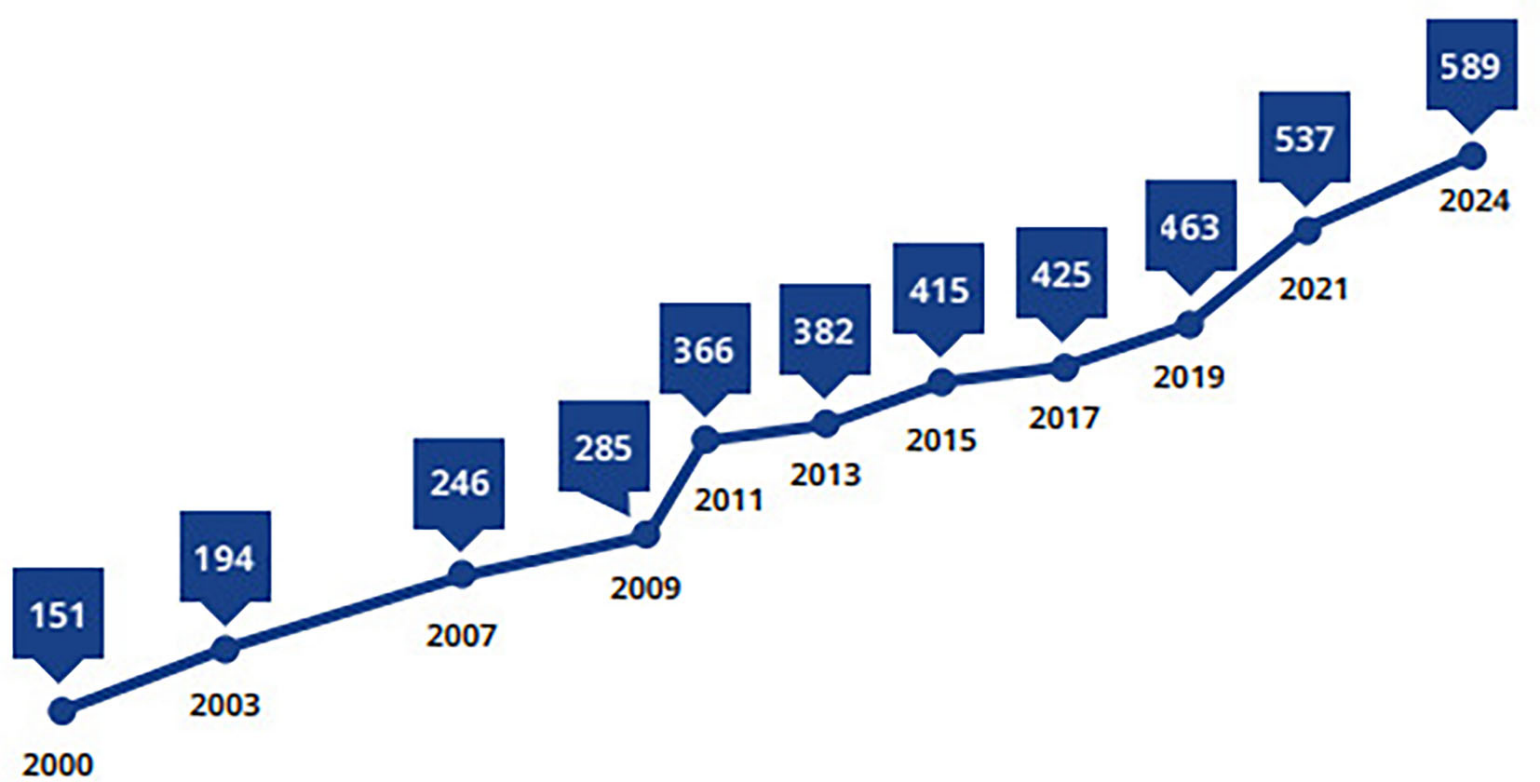
The number of people diagnosed with DM as presented in millions. ^1^

Diabetes mellitus (DM) is one of the most common chronic diseases in the world characterized by carbohydrate metabolism disorders. ^1^ There were approximately 589 million people aged 20 to 79 years suffering from DM in 2025 that approximately 11.1% of the population as shown in Figure 1. ^1, 2^ Diabetes Mellitus (DM) can be caused by disorders in insulin production, cells resistance to insulin, or both. This chronic hyperglycemic condition can interact with other metabolic problems in people with DM causing organ damage and resulting in serious complications. ^1, 2^ These complications include microvascular such as retinopathy, nephropathy, and neuropathy, as well as macrovascular causing coronary arteries, peripheral arteries, and cerebrovascular diseases. ^3^

Long-term complications of DM can cause serious health problems, one of which is sexual dysfunction in men and women. In men, sexual dysfunction due to DM is Erectile Dysfunction (ED) with a prevalence of 3.5 times higher than in men without DM. ^4^ Erectile Dysfunction (ED) in men with DM is also associated with decreased fertility in men due to hypothalamic-pituitary-gonadal axis dysfunction, spermatogenesis and maturation disorders. ^5^

Erectile Dysfunction (ED) and reproductive disorders in men are related to testosterone level. Many studies have shown that testosterone deficiency is common in men with DM, both Type 1 DM (T1DM) and Type 2 DM (T2DM). In people with T2DM, there is a decrease in free testosterone of up to 57%, while in people with T1DM, the decrease in free testosterone reaches 20.3%. Thus, it is not an exaggeration to say that total testosterone and free testosterone levels are risk factors for DM in men. ^6, 7^

Apart from testosterone as a risk factor for DM, low testosterone levels themselves are known to cause decreased sexual function and fertility in men. The low of sexual function is characterized by loss of erection, decreased sexual desire, and decreased frequency of sexual intercourse. ^8^ Research on animal model has shown that testosterone is also a determining factor in male fertility because it affects spermatogenesis. The critical processes in spermatogenesis that are influenced by testosterone are maintaining the blood testes barrier, supporting the meiosis process, the adhesion of spermatids to Sertoli cells, and the release of sperms. ^9^

Currently, there are many types of drugs commonly used to treat DM, including metformin, sulfonylureas, meglitinides, thiazolidinediones, dpp-4 inhibitors. Unfortunately, all of these drugs have side effects. Metformin, for example, causes side effects such as nausea, vomiting, abdominal bloating, diarrhea, heartburn, headache, agitation, dizziness, tiredness, chills, abdominal cramps or pain, loss of appetite, asthenia, and myalgia. ^10^ Therefore, the search for DM drugs derived from plants continues to grow. One of the medicinal plants that has the potential to have anti-diabetic properties is black pepper ( *Piper nigrum L.*). Tests on alloxan-induced diabetic rats showed that black pepper fruit extract was effective in lowering blood sugar levels and was also effective in lowering cholesterol levels. ^11, 12^

Apart from having the potential as an anti-diabetic, black pepper fruit extract has also been proven to increase testosterone hormone levels, sexual function (libido), and spermatogenesis parameters in male rats. Mating tests on male rats given black pepper fruit extract showed a significant increase in libido, indicated by a shorter courtship latency. ^13^ The fertility parameters of male rats given black pepper fruit extract also increased, indicated by an increase in epididymal sperm concentration, spermatocyte counts, spermatid counts, and the weight of epididymis tubules. ^14^

Although black pepper has shown antidiabetic and reproductive benefits, its effect on DM-related ED is not well established. Especially, its influence on erectile function, libido parameters, spermatozoa parameters, and testicular histology parameters has not been evaluated. To our knowledge, this is the first to demonstrate the potential of black pepper ( *Piper nigrum L.*) fruit extract to improve sexual function as depicted in libido parameters, spermatozoa parameters, and testicular histology parameters in alloxan-induced diabetic rats.

## Materials and methods

### Study design

This study was an experimental, randomized controlled laboratory to assess the effects of black pepper ( *Piper nigrum L.*) fruit extract on libido parameters, spermatozoa parameters, and testicular histology parameters in alloxan-induced diabetic rats. Rats were randomly sampled into control, diabetic, extract-treated, and positive control groups to allow direct comparison of treatment outcomes. Data obtained were collected and analyzed using the IBM SPSS Statistics version 31 software (New York, USA). Normality of the data was assessed with the Shapiro-Wilk test. Since the data was considered non-parametric, Kruskal-Wallis tests were used. Differences between groups were evaluated using One-Way Analysis of Variance (ANOVA), followed by a Least Significant Difference (LSD) post hoc test to identify specific intergroup differences. A *p*-value of <0.05 was considered statistically significant.

### Plant preparation

Fresh black pepper fruit is collected from a farmer in Ngarip Village, Ulubelu District, Tanggamus Regency, Lampung Province. The fresh fruit is washed and rinsed until clean and then dried. After drying, the fruit is ground into powder using a blender. The extraction was done by soaking the black pepper powder in 95% ethanol at room temperature. The supernatant collected every 24 h for three days and filtered to remove unwanted components. The filtrate was concentrated using a rotary evaporator at a temperature of 40°C and a pressure of 60 mbar. The extract is stored in the refrigerator as stock until used.

### Animal preparation

This study used 30 male Sprague Dawley rats as mentioned by Federer study before, Rattus norvegicus, aged 2.5-3 months weighing 100-150 grams obtained from the animal house at the IPB University, Bogor, Indonesia. All procedures were conducted in accordance and approved with the guidelines of the Health Research Ethics Commission of the Faculty of Medicine, Lampung University (No.3468/UN26.18/PP.05.02.00/2024). The rats were acclimated for a week at a temperature of 25°C with stable room humidity and lighting, and were given standard feed ad libitum.

The studied rats were divided into five groups of 6 individuals each. Group 1 (I) was rats that were only given standard feed. Group 2 (II) was alloxan-induced hyperglycemic rats and given feed. Groups 3 (III) and group 4 (IV) were alloxan-induced hyperglycemic rats and given black pepper extract 122.5 and 245 mg/kg BW respectively for 8 days. Group 5 (V) is alloxan-induced hyperglycemic rats given Sildenafil therapy.

Alloxan induction in studied rats was performed intraperitoneally using normal saline solvent at a dose of 150 mg/kg BW. To prevent alloxan-induced rats from suffering from severe hypoglycemia, the rats were given a 20% glucose solution (5-10 ml) orally after 6 hours. Furthermore, after being maintained for 24 hours, the rats were given further 5% glucose solution to prevent hypoglycemia. Rats are categorized to have DM if they experience glycosuria and hyperglycemia with blood glucose levels of 200 to 300 mg/dL. ^15^

Phosphodiesterase-5 inhibitor (PDE-5i) therapy was given one hour before observation of erectile function and libido assessment. The therapy was done using Sildenafil Citrate, a drug from the PDE-5i at a dose of 1 mg/kg BW as positive control for recovery of ED caused by DM. ^16^ Sildenafil treatment was done following Gurbuz et al. (2015) to decrease advanced glycation end products, Malondialdehyde (MDA) and Inducible Nitric Oxide Synthase (iNOS), to preserve Neuronal Nitric Oxide Synthase (nNOS) and Cyclic Guanosine Monophosphate (cGMP) contents in penile tissue. ^17^

### Erectile function assessment

Erectile function of the rats was assessed on the ninth day one hour before the mating test was performed. The animals were placed in supine position with partial restraint. The preputial sheath was pushed behind the gland penis and held in this manner for a period of 15 min to elicit penile reflex. Total Penile Reflexes (TPR) for each animal were calculated as the sum of Erection (E), Long Flips (LF) and Quick Flips (QF). Mathematically, it can be expressed using the following formula TPR = E+QF+LF. ^18, 19^

### Libido assessment

To determine the libido level of the studied rats, a mating test was conducted on the ninth day. The mating test was conducted by placing male and female rats in a cage with a partition in the middle. The cage was placed in a room with low lighting. Observation and analysis of the mating behavior of rats were conducted through video recording. There were three mating behavior parameters assessed, namely courtship latency, mounting latency, and mounting frequency. Courtship latency is the time from the partition being opened until the male kisses the female’s genitals or other body parts. Mounting latency is the time from the partition being opened until the male mounts the female’s back. Mounting frequency is the number of times the male mounts the female’s back during the 30 minutes (1800 seconds) of the mating test.

### Sperm analysis and testis histology examination

After the erectile function and mating behavior were assessed, sperm and histological analysis of the studied rats were performed. The sperm analysis and testis histology examination were performed in Anatomy Pathology Laboratorium of Faculty of Medicine, Lampung University. The rats were terminated by injection of ketamine at a dose of 100 mg/kg BW and cervical dislocation. A total of 30 right testes and cauda epididymis were removed using a dissecting kit. The cauda epididymis were pinched to squeeze the spermatozoa into the petri dish. Subsequently, a suspension of spermatozoa were formed after being mixed with NaCL 0.9%, as mentioned in previous study. ^20^ Spermatozoa were counted using a Neubauer’s hemocytometer under a light microscope (400x) and expressed as millions/ml. Sperm motility was evaluated by counting motile and immotile sperms. Sperm morphology was assessed from a smear of the epididymal filtrate prepared on a clean glass slide with 1% eosin staining. After the object dried, observation was done under Richter Optica HS-3B-3’s light microscope (China) at 400x magnification and abnormalities of either head or tail were noted. Spermatozoa parameters were only assessed in studied rats of group 1 to group 4. Testicular histology examination was performed using Haematoxylin & Eosin (HE) staining and spermatogonia were counted in 10 random seminiferous tubule cross-sections per testis, and Leydig cells were counted in 10 non-overlapping interstitial fields with thickness of 4-5 μm; counts were normalized to area using National Institute of Health ImageJ software version 1.8.0 (New York, USA) (calibrated to μm ^2^) at 400x magnification. ^21^

## Results

### Erectile function

Erectile function parameters of alloxan-induced rats treated with black pepper fruit extract are presented in Table 1. Group I, consisting of normal rats with normal feeds, showed the highest average values for the quick flip and erection parameters. As the reference group in this study, Group I also had the highest average TPR values compared to the other groups. Among the alloxan-induced rats, Group III (treated with black pepper extract at a dose of 122.5 mg/kg BW) demonstrated average quick flip, long flip, and erection values that were comparable to those of Group I. The average TPR value in Group III was also similar to that of Group I. However, Group IV, which received a higher dose of black pepper extract (245 mg/kg BW), showed relatively lower average values for quick flip, long flip, and erection compared to Group III, with TPR values more comparable to those of Group II. As shown in Table 1, Group III had relatively higher average TPR values compared to Group V. The treatment effect was observed only in the TPR parameter, whereas the differences in ER, QF, and LF parameters were not statistically significant. Diabetic rats given black pepper fruit extract at a dose of 122.5 mg/kg BW (Group III) showed a statistically significant differences in TPR values compared to alloxan-induced rats with normal feeds (Group II).

**Table 1.** Erectile function parameters of studied rats.

Group	QF	LF	ER	TPR	TPR *p*-value ^#^
I (only fed)	3,67 ± 0,82 ^a^	1.33 ± 0.52 ^a^	4.67 ± 0.82 ^a^	9.67 ± 1.03 ^a^	vs II = **<0.001**; vs III = 0.246; vs IV = 1.000; vs V = 0.135
II (alloxan)	1.83 ± 0.75 ^a^	0.33 ± 0.52 ^a^	3.83 ± 0.75 ^a^	6.00 ± 1.26 ^a^	vs I = **<0.001**; vs III = **0.02**; vs IV = < **0.001**; vs V = **0.038**
III (extract 122.5 mg/kg BW)	3.33 ± 1.03 ^a^	1.67 ± 0.52 ^a^	4.33 ± 0.52 ^a^	9.33 ± 1.03 ^a^	vs I = 0.246; vs II = **0.02**; vs IV = 0.747; vs V = 1.000
IV (extract 245 mg/kg BW)	2.83 ± 0.75 ^a^	0.67 ± 0.82 ^a^	3.33 ± 0.82 ^a^	6.83 ± 0.98 ^a^	vs I = 1.000; vs II = **<0.001**; vs III = 0.747; vs V = 0.436
V (sildenafil citrate)	2.17 ± 0.75 ^a^	1.33 ± 0.52 ^a^	4.67 ± 0.52 ^a^	8.17 ± 1.17 ^a^	vs I = 0.135; vs II = **0.038**; vs III = 1.000; vs IV = 0.436

QF = Quick Flip, LF = Long Flip, E = Erection, TPR = Total Penile Reflexes,

^a^ = Presented in mean and Standard Deviation (SD),

^#^ = LSD Post Hoc test.

### Libido

The effect of giving black pepper extract on the libido of rats using the parameters of courtship latency, mounting latency and mounting frequency can be seen in Table 2. Based on the data in Table 2, it can be seen that the administration of black pepper fruit extract significantly improved the decreased libido due to hyperglycemia caused by alloxan induction. Based on the values of the courtship latency, mounting latency and mounting frequency, it was revealed that the best dose of black pepper extract to restore the libido of hyperglycemic male rats was a dose of 122.5 mg/kg BW. The therapeutic effect with sildenafil citrate also significantly improved the libido of rats.

**Table 2.** Libido parameters of Courtship Latency (CL), and Mount Latency (ML) of studied rats.

Group	CL	CL *p*-value ^^^	ML	ML *p*-value ^#^
I (only fed)	5.17 ± 0.41 ^a^	vs II = **0.003**; vs III = 0.060; vs IV = 0.241; vs V = **0.005**	8.50 ± 3.51 ^a^	vs II = **<0.001**; vs III = **0.033**; vs IV = **0.006**; vs V = **<0.001**
II (alloxan)	21.00 ± 9.47 ^a^	vs I = **0.003**; vs III = **0.013**; vs IV = **0.003**; vs P2 = **0.003**	37.17 ± 6.31 ^a^	vs I = **<0.001**; vs III = **0.009**; vs IV = **<0.001**; vs V = 0.075
III (extract 122.5 mg/kg BW)	5.50 ± 0.55 ^a^	vs I = 0.06; vs II = **0.013**; vs IV = 0.204; vs V = 1.000	19.00 ± 10.81 ^a^	vs I = **0.033**; vs II = **0.009**; vs IV = 0.464; vs V = **0.007**
IV (extract 245 mg/kg BW)	7.00 ± 1.10 ^a^	vs I = 0.241; vs II = **0.003**; vs III = 0.204; vs V = **0.019**	27.67 ± 6.53 ^a^	vs I = **0.006**; vs II = **<0.001**; vs III = 0.464; vs V = **0.026**
V (sildenafil citrate)	9.00 ± 4.82 ^a^	vs I = **0.005**; vs II = **0.003**; vs III = 1.000; vs IV = **0.019**	16.00 ± 8.27 ^a^	vs I = **<0.001**; vs II = 0.075; vs III = **0.007**; vs IV = **0.026**

CL = Courtship Latency, ML = Mount Latency,

^a^ = Presented in mean and Standard Deviation (SD),

^^^ = Mann-Whitney test,

^#^ = LSD Post Hoc test.

Courtship Latency (CL) and Mount Latency (ML) showed significant differences among groups as shown in Table 2 and Table 3. The alloxan group (II) had the longest CL (21.00 ± 9.47, p = 0.003 vs group I) and ML (37.17 ± 6.31, p < 0.001 vs group I). In contrast, the extract 122.5 mg/kg BW group (III) demonstrated CL (5.50 ± 0.55) and ML (19.00 ± 10.81), both significantly different from group II (p = 0.013 and p = 0.009, respectively) and closer to group I. The extract 245 mg/kg BW group (IV) also showed shorter CL (7.00 ± 1.10) and ML (27.67 ± 6.53) compared with group II (p = 0.003 and p < 0.001, respectively). The sildenafil group (V) exhibited CL (9.00 ± 4.82) and ML (16.00 ± 8.27), with significant differences from group II (p = 0.003 and p = 0.075, respectively).

**Table 3.** Mount Frequency (MF) of studied rats.

Group	MF	MF *p*-value ^#^
I (only fed)	17.00 ± 3.74 ^a^	vs II = **<0.001**; vs III = 0.351; vs IV = 0.649; vs V = 0.157
II (alloxan)	7.17 ± 1.83 ^a^	vs I = **<0.001**; vs III = **0.002**; vs IV = **<0.001**; vs V = **0.006**
III (extract 122.5 mg/kg BW)	18.05 ± 5.99 ^a^	vs I = 0.351; vs II = **0.002**; vs IV = 0.17; vs V = 0.615
IV (extract 245 mg/kg BW)	13.33 ± 3.45 ^a^	vs I = 0.649; vs II = **<0.001**; vs III = 0.17; vs IV = 0.066
V (sildenafil citrate)	14.83 ± 5.74 ^a^	vs I = 0.157; vs II = **0.006**; vs III = 0.615; vs IV = 0.066

MF = Mount Frequency,

^a^ = Presented in mean and Standard Deviation (SD),

^#^ = LSD Post Hoc test.

Mount Frequency (MF) was lowest in group II (7.17 ± 1.83), significantly different from group I (17.00 ± 3.74, p < 0.001). Group III (18.05 ± 5.99) was not significantly different from group I (p = 0.351), while group IV (13.33 ± 3.45) and group V (14.83 ± 5.74) showed higher MF compared with group II (p < 0.001 and p = 0.006, respectively).

### Sperm analysis

In sperm concentration (SC), the control group (I) recorded a mean of 75.81 ± 52.9, which was significantly higher than group II (12.6 ± 1.3; p = 0.002) and group III (19.2 ± 6.7; p = 0.004), but not different from group IV (62.95 ± 29.4; p = 0.483) as shown in Table 4. Compared with group V (158.16 ± 29.8), group I was significantly lower (p < 0.001). Group II had significantly lower SC compared with group IV (p = 0.01) and group V (p < 0.001), but no significant difference from group III (p = 0.877). Group III showed significantly lower SC than group IV (p = 0.019) and group V (p < 0.001). Group IV exhibited significantly lower SC than group V (p < 0.001). Overall, group V demonstrated the highest SC among all groups with significant differences in all comparisons (p < 0.001).

**Table 4.** Sperm analysis parameters of sperm concentration, and sperm progressive motility of studied rats.

Group	SC	SC *p*-value ^#^	SPM	SPM *p*-value ^#^
I (only fed)	75.81 ± 52.9 ^b^	vs II = **0.002**; vs III = 0.004*; vs IV = 0.483; vs V = **<0.001**	57 ± 33 ^a^	vs II = **0.024**; vs III = 0.07; vs IV = 0.121; vs V = 0.556
II (alloxan)	12.6 ± 1.3 ^a^	vs I = **0.002**; vs III = 0.877; vs IV= **0.01**; vs V = **<0.001**	27 ± 30 ^a^	vs I = **0.024**; vs III = 0.697; vs IV = 0.436; vs V = **0.006**
III (extract 122.5 mg/kg BW)	19.2 ± 6.7 ^a^	vs I = **0.004**; vs II = 0.877; vs IV = **0.019**; vs V = **<0.001**	31.8 ± 23 ^a^	vs I = 0.07; vs II = 0.697; vs IV = 0.721; vs V = **0.021**
IV (extract 245 mg/kg BW)	62.95 ± 29.4 ^a^	vs I = 0.483; vs II = **0.01**; vs III = **0.019**; vs V = < **0.001**	36.6 ± 23 ^a^	vs I = 0.121; vs II = 0.436; vs III = 0.721; vs V = **0.037**
V (sildenafil citrate)	158.16 ± 29.8 ^a^	vs I = **<0.001**; vs II = **<0.001**; vs III = **<0.001**; vs IV = **<0.001**	65 ± 35 ^a^	vs I = 0.556; vs II = 0.006*; vs III = **0.021**; vs IV = **0.037**

SC = Sperm Concentration, SPM = Sperm Progressive Motility,

^a^ = Presented in mean and Standard Deviation (SD),

^#^ = LSD Post Hoc test.

For sperm progressive motility (SPM) as shown in Table 4, the control group (I) recorded 57 ± 33, which was significantly higher than group II (27 ± 30; p = 0.024), but not different from group III (31.8 ± 23; p = 0.07) or group IV (36.6 ± 23; p = 0.121). Compared with group V (65 ± 35), no significant difference was observed (p = 0.556). Group II showed significantly lower SPM than group V (p = 0.006) but not significantly different from group III (p = 0.697) or group IV (p = 0.436). Group III had significantly lower SPM compared with group V (p = 0.021) but did not differ significantly from group IV (p = 0.721). Group IV also showed significantly lower SPM than group V (p = 0.037). Thus, group V demonstrated the highest SPM, with significant superiority over groups II–IV.

Regarding sperm normal morphology (SNM) as presented in Table 5, the control group (I) recorded 75 ± 13.7, which was significantly higher than group II (35 ± 10.8; p < 0.001) and group III (40.9 ± 7.8; p < 0.001), but not different from group IV (62 ± 14.7; p = 0.119) or group V (82.9 ± 5.7; p = 0.273). Group II had significantly lower SNM than all other groups (p ≤ 0.04). Group III also showed significantly lower SNM compared with group IV (p = 0.017) and group V (p < 0.001). Group IV exhibited significantly lower SNM than group V (p = 0.011). Among all groups, group V demonstrated the highest SNM, with significant differences against groups II–IV.

**Table 5.** Sperm analysis parameters of sperm normal morphology of studied rats.

Group	SNM	SNM *p*-value ^#^
I (only fed)	75 ± 13.7 ^a^	vs II = **<0.001**; vs III = **<0.001**; vs IV = 0.119; vs V = 0.273
II (alloxan)	35 ± 10.8 ^a^	vs I = **<0.001**; vs III = **0.04**; vs IV = **<0.001**; vs V = < **0.001**
III (extract 122.5 mg/kg BW)	40.9 ± 7.8 ^a^	vs I = **<0.001**; vs II = **0.04**; vs IV = **0.017**; vs V = **<0.001**
IV (extract 245 mg/kg BW)	62 ± 14.7 ^a^	vs I = 0.119; vs II = **<0.001**; vs III = **0.017**; vs V = **0.011**
V (sildenafil citrate)	82.9 ± 5.7 ^a^	vs I = 0.273; vs II = **<0.001**; vs III = **<0.001**; vs IV = **0.011**

SNM = Sperm Normal Morphology,

^a^ = Presented in mean and Standard Deviation (SD),

^#^ = LSD Post Hoc test.

### Testicular histology analysis

**Figure 2.**
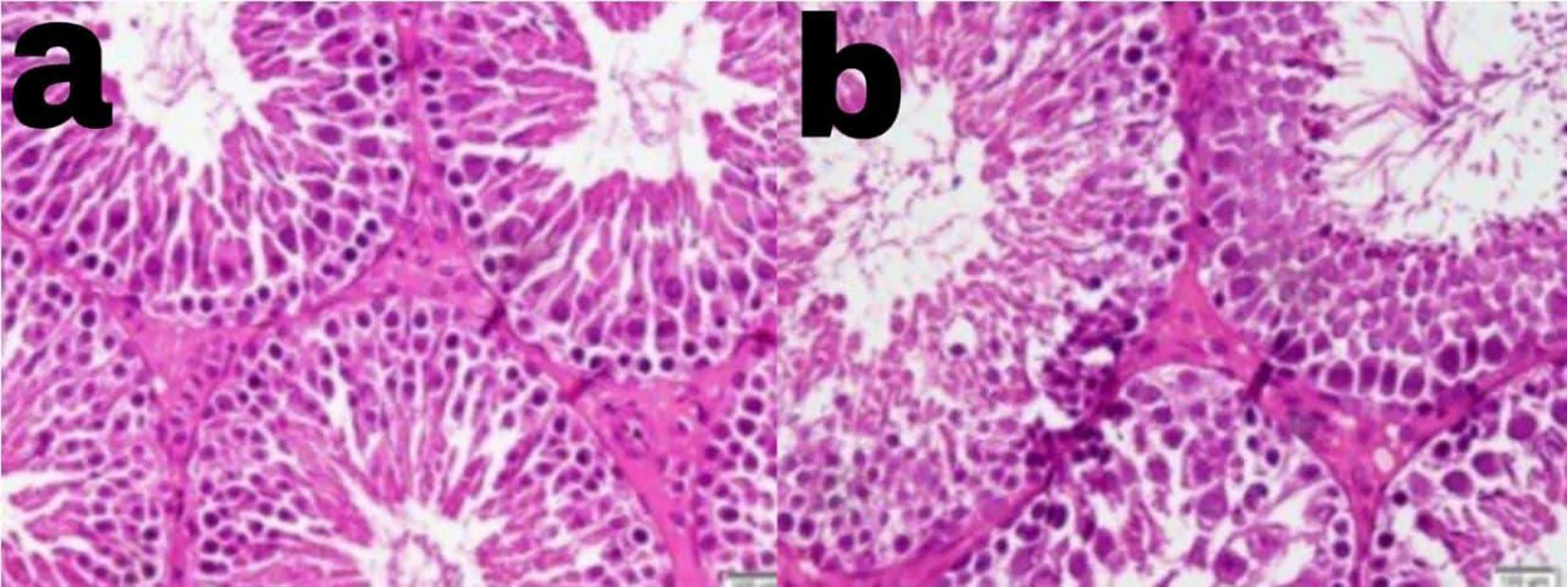
Higher amounts of spermatogonia was observed in group III with black pepper extract 122.5 mg/kg BW (a) as compared in group II without black pepper extract (b).

Table 6 presents the examination results of the testicular histology of alloxan-induced diabetic rats treated with black pepper extract. The data in the table shows that black pepper extract at a dose of 122.5 mg/kg BW significantly restored the number of Leydig cells and spermatogonia count. As shown in Figure 2, group III with black pepper extract of 122.5mg/kg BW showed a higher amounts of spermatogonia as compared with group II without black pepper extraction.

**Table 6.** Testicular histology parameters.

Group	LC	LC *p*-value	SgC	SgC *p*-value
I (only fed)	60.83 ± 5.1 ^a^	vs II = **<0.001**; vs III = **<0.001**; vs IV = 0.581; vs V = **0.001**	511 ± 73.4 ^a^	vs II = 0.593; vs III = **<0.001**; vs IV = 0.294; vs V = **0.013**
II (alloxan)	30.50 ± 3.86 ^a^	vs I = **<0.001**; vs III = **0.035**; vs IV = **<0.001**; vs V = **0.001**	491 ± 37.0 ^a^	vs I = 0.593; vs III = **<0.001**; vs IV = 0.119; vs V = **0.042**
III (extract 122.5 mg/kg BW)	59.33 ± 4.0 ^a^	vs I = **<0.001**; vs II = **0.035**; vs IV = **<0.001**; vs V = **<0.001**	319.4 ± 64.59 ^a^	vs I = **<0.001**; vs II = **<0.001**; vs IV = **<0.001**; vs V = **0.014**
IV (extract 245 mg/kg BW)	50.67 ± 2.73 ^a^	vs I = 0.581; vs II = **<0.001**; vs III = **<0.001**; vs IV = **0.003**	640 ± 86.5 ^a^	vs I = 0.294; vs II = 0.119; vs III = **<0.001**; vs V = **0.001**
V (sildenafil citrate)	32.60 ± 3.28 ^a^	vs I = **0.001**; vs II = **<0.001**; vs III = **<0.001**; vs P1 = **0.003**	414 ± 32.5 ^a^	vs I = **0.013**; vs II = **0.042**; vs III = **0.014**; vs IV = **0.001**

LC = Leydig Cell Count, SgC = Spermatogonium Cell Count,

^a^ = Presented in mean and Standard Deviation (SD),

^#^ = LSD Post Hoc test.

## Discussion

**Figure 3.**
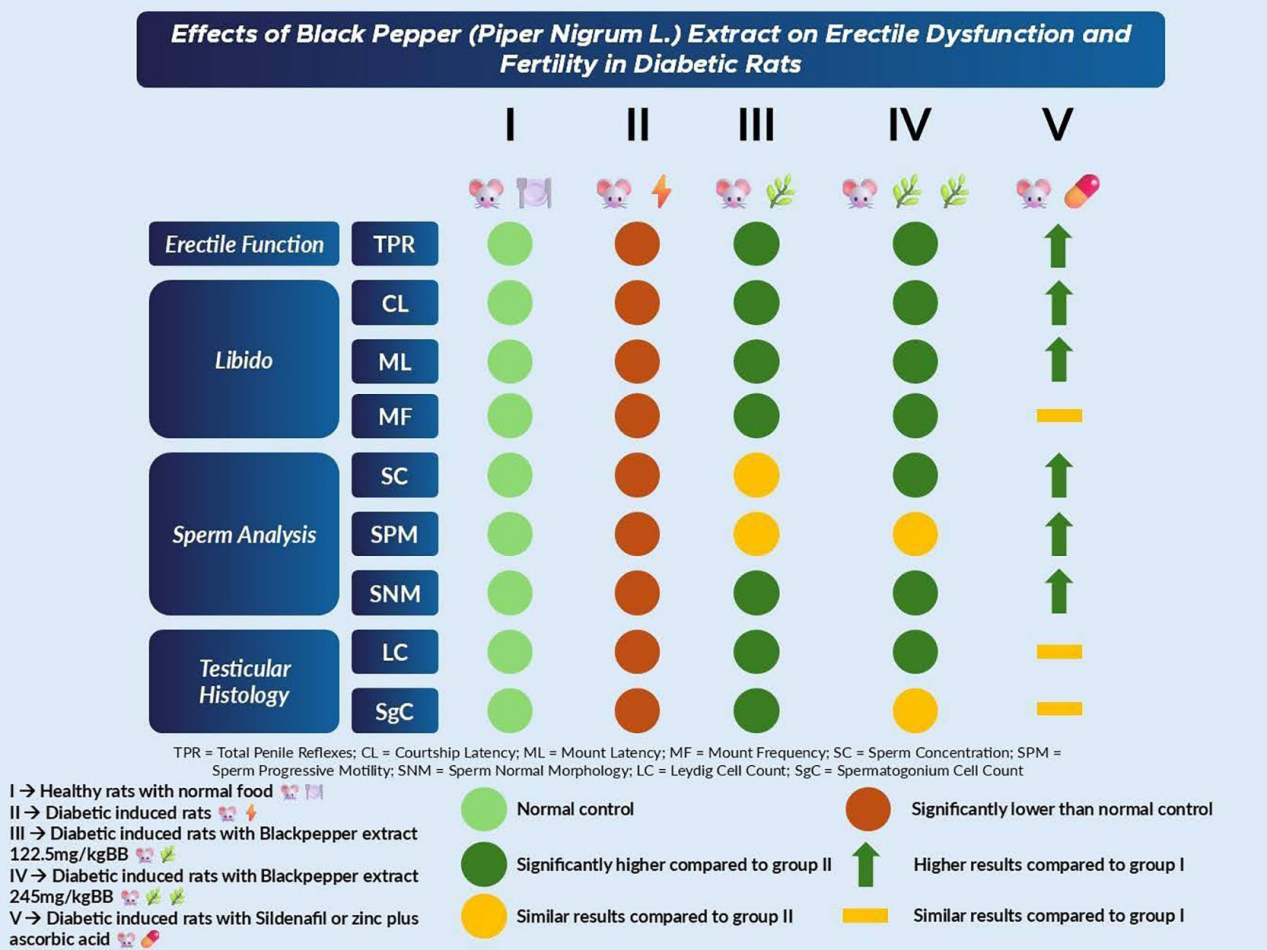
Summary of results.

The present study demonstrated that administration of *Piper nigrum* fruit extract ameliorated reproductive parameters in alloxan-induced diabetic male rats, as evidenced by increased sperm concentration, motility, serum testosterone levels, and improved morphology of seminiferous tubules. These findings are consistent with previous reports showing that black pepper extract can enhance fertility potential and improve reproductive outcomes in diabetic or hyperglycemic animal models, most likely through attenuation of oxidative stress and restoration of metabolic status. ^20, 21^ In Figure 3, we presented the summary of our results based on erectile function, libido, sperm analysis, and testicular histology.

The decline in erectile function in alloxan-induced diabetic rats may be related to insulin resistance. Insulin resistance is known to be the cause of erectile dysfunction and hypogonadism. ^22^ Persistent hyperglycemia triggers Endoplasmic Reticulum Stress (ERS) in Leydig cells of the testis, causing increased expression of ERS-related factors, resulting in Leydig cell apoptosis and disrupting testosterone production, resulting in decreased erectile function. ^23, 24^ Insulin deficiency in diabetes mellitus also causes excessive secretion of arginase II in the corpus cavernosum so that Nitric Oxide (NO) cannot function properly, resulting in inadequate blood flow to the penis. This can trigger erectile dysfunction. ^25, 26^

This study revealed that alloxan (Group II) induction significantly decreased libido parameters in male rats. This occurred, according to various reports, because hyperglycemic conditions caused severe gonadal dysfunction, reproductive dysfunction, and diminished body and reproductive and organ weight. ^27^

Male rats with alloxan-induced hypoglycemia treated with black pepper extract were found to have improved sexual function. This recovery is very likely due to the influence of piperine in black pepper fruit. Piperine is an active ingredient that is typically found in Piperaceae plants. Piperine extracted from the fruit plant of Piper retrofractum L. plant, for example, is known to have an aphrodisiac effect on the libido of male rats. ^28^

The restoration of sexual function by black pepper fruit extract is related to the effect of piperine which can inhibit the work of testosterone 5"-reductase so that testosterone levels remain high. In addition, black pepper fruit extract is also known to contain fatty acids, such as auric acid, myristic acid and palmitic acid which affect androgen secretion and metabolism. Black pepper fruit extract is also known to contain zinc which promotes increased sex hormones, including testosterone in serum. ^29^ Testosterone is a hormone that has been proven to increase sexual desire or libido in hypogonadal men (patients). ^30^

This study shows that alloxan induction in male rats (Group II) causes a significant decrease in spermatozoa concentration and Leydig cell count compared to normal rats (Group I). This may be related to endocrine imbalance and impaired glucose transport and metabolism in diabetic rats. Endocrine imbalance – dysfunction of HPG axis occurs in diabetic patients which can cause infertility. Hyperglycemia exposure causes decreased sensitivity of the pituitary to gonadotropin-releasing hormone (GnRH) stimulation, resulting in abnormal secretion of FSH and LH. FSH plays a role in stimulating testicular Sertoli cells to promote sperm maturation in the seminiferous tubules, LH plays a role in stimulating Leydig cells in increasing serum testosterone levels. This decreased sensitivity of pituitary to GnRH causes disorders in the male reproductive system. ^30^

This research showed that administration of black pepper fruit extract at a dose of 245 mg/kg to alloxan-induced diabetic rats significantly increased the number and motility of spermatozoa, but had no effect on the abnormal morphology of the spermatozoa. On the other hand, administration of black pepper extract at a concentration of 122.5 mg/kg significantly increased the number of Leydig cells and the number of spermatogonia. This result is not surprising because the study reported by Chinta et al. (2017) showed that administration of piperine to rats causes hormonal imbalance by altering the serum levels of follicle-stimulating hormone, luteinizing hormone, sex hormone binding globulin, serum, and testicular testosterone. ^31^

Other research results showing piperine effects that are in line with the findings of this study were reported by Chen et al. (2018). Piperine increase serum testosterone, increased Leydig cell number, cell size, multiple steroidogenic pathway proteins, 3β-hydroxysteroid dehydrogenase 1, 17α-hydroxylase/20-lyase, and steroidogenic factor 1 expression levels. Piperine stimulates pubertal Leydig cell development by increasing the number of Leydig cell and promoting its maturation. However, because piperine reduce FSH, then it inhibits spermatogenesis in. ^32^ Other researcher (Ere et al., 2020) reported that administration of black pepper fruit extract to rabbits increased testicular morphometric parameters, but caused damage to the seminiferous tubules and decreased spermatogenesis. ^33^

## Conclusion

Administration of black pepper fruit extract to male alloxan-induced diabetic rats significantly increased erectile function, libido, spermatozoa count, and Leydig cell count and spermatogonia count. Therefore, it can be concluded that black pepper extract ( *Piper nigrum* L.) has potential as an ingredient to overcome sexual dysfunction and reproductive dysfunction in males. However, this topic still an early study, we still need bigger and randmoized sample, especially human subject.

## Data Availability

Figshare: Black pepper (Piper nigrum L.) fruit extract ameliorates erectile dysfunction in alloxan-induced diabetic rats. Available at: https://doi.org/10.6084/m9.figshare.30456353. ^34^ The project contains the following underlying data:
• Erectile function parameters values on each group • Libido parameters values on each group • Sperm analysis parameters values on each group • Testicular histology parameters values on each group Figshare: Black pepper (Piper nigrum L.) fruit extract ameliorates erectile dysfunction in alloxan-induced diabetic rats. Available at: https://doi.org/10.6084/m9.figshare.30456353. ^34^ The project contains the following underlying data:
• qPCR eNOS values on the subjects • ARRIVE guidelines - Author checklist
