# Effects of Black Pepper (Piper nigrum L.) Fruit Extract on Sexual Function, Hormonal Parameters, Sperm Parameters, Testicular Histology, and eNOS Expression in Alloxan-Induced Diabetic Rats

**DOI:** 10.12688/f1000research.172637.2

**Published:** 2026-04-29

**Authors:** Exsa Hadibrata, Sutyarso Sutyarso, Hendri Busman, Syazili Mustofa, Wawan Abdullah Setiawan, Ratna Dewi Puspita Sari, Nuning Nurcahyani

**Affiliations:** 1 Doctoral Program, Universitas Lampung Fakultas Matematika dan Ilmu Pengetahuan Alam, Bandar Lampung, Lampung, Indonesia; 2 Department of Surgery, Universitas Lampung Fakultas Kedokteran, Bandar Lampung, Lampung, Indonesia; 3 Department of Biology, Universitas Lampung Fakultas Matematika dan Ilmu Pengetahuan Alam, Bandar Lampung, Lampung, Indonesia; 4 Department of Biochemistry and Biomolecular, Universitas Lampung Fakultas Kedokteran, Bandar Lampung, Lampung, Indonesia; 5 Department of Obstetric and Gynecology, Universitas Lampung Fakultas Kedokteran, Bandar Lampung, Lampung, Indonesia

**Keywords:** Diabetes Mellitus, Hyperglycemia, Black Pepper, Piper Nigrum, Erectile Function, Libido

## Abstract

**Background:**

Diabetes mellitus (DM) is often associated with male sexual and reproductive dysfunction, including erectile impairment, reduced libido, hormonal disturbances, and poor semen quality. Black pepper ( *Piper nigrum* L.) fruit extract has shown antidiabetic and reproductive benefits in experimental studies, but its role in diabetes-related sexual dysfunction remains unclear.

**Objective:**

To evaluate the effects of black pepper fruit extract on hormonal, erectile, libido, sperm, histological, and molecular parameters in alloxan-induced diabetic male rats.

**Methods:**

Thirty male Sprague Dawley rats were assigned to five groups: normal control, diabetic control, diabetic rats treated with black pepper extract at 122.5 or 245 mg/kg body weight (BW) for 8 consecutive days, and diabetic rats treated with sildenafil citrate 1 mg/kg BW. Outcomes included intratesticular testosterone (ITT), erectile parameters (quick flip, long flip, erection, total penile reflexes [TPR]), libido parameters (courtship latency [CL], mount latency [ML], mount frequency [MF]), sperm parameters (concentration, progressive motility, normal morphology), Leydig cell and spermatocyte counts, and eNOS mRNA expression. Data were analyzed using the Kruskal-Wallis test followed by Dunn’s post hoc test.

**Results:**

Compared with diabetic controls, the 122.5 mg/kg BW black pepper group significantly improved TPR, CL, ML, and MF. The 245 mg/kg BW group significantly improved ITT, sperm concentration, and normal sperm morphology. Both black pepper doses increased Leydig cell counts. Sildenafil significantly improved TPR, sperm concentration, progressive motility, and normal morphology. eNOS mRNA expression was higher in all treatment groups than in diabetic controls.

**Conclusion:**

Black pepper fruit extract showed dose-dependent effects in alloxan-induced diabetic rats. The lower dose was more beneficial for erectile and libido-related parameters, whereas the higher dose had greater effects on ITT and selected sperm outcomes. These findings support its potential as a preclinical candidate for diabetic male sexual and reproductive dysfunction.

## Introduction

Diabetes mellitus (DM) is one of the most common chronic diseases in the world characterized by carbohydrate metabolism disorders. ^1^ The most recent epidemiological data from the International Diabetes Federation (IDF) Diabetes Atlas 11th edition, published in 2025, estimate that 589 million adults aged 20–79 years were living with diabetes worldwide in 2024, representing 11.1% of the global adult population. Type 2 diabetes mellitus (T2DM) accounts for approximately 90–95% of all diabetes cases, making it the predominant form of the disease globally. Moreover, the number of adults living with diabetes is projected to increase to 853 million by 2050, representing the peak projected global burden based on current IDF estimates. ^2^ Diabetes Mellitus (DM) can be caused by disorders in insulin production, cells resistance to insulin, or both. This chronic hyperglycemic condition can interact with other metabolic problems in people with DM causing organ damage and resulting in serious complications. ^1^ ^,^ ^2^ These complications include microvascular such as retinopathy, nephropathy, and neuropathy, as well as macrovascular causing coronary arteries, peripheral arteries, and cerebrovascular diseases. ^3^

Long-term complications of DM can cause serious health problems, one of which is sexual dysfunction in men and women. In men, sexual dysfunction due to DM is Erectile Dysfunction (ED) with a prevalence of 3.5 times higher than in men without DM. ^4^ Erectile dysfunction in men with diabetes is a multifactorial disorder involving vascular, neural, endocrine, and structural mechanisms. ^5^ Chronic hyperglycemia promotes oxidative stress and inflammation, which impair endothelial function and reduce nitric oxide bioavailability, thereby disturbing cavernosal smooth muscle relaxation and penile blood flow required for erection. ^6^ In addition, diabetes-associated autonomic and peripheral neuropathy may disrupt the neural signaling necessary for the initiation and maintenance of erection. ^5^ Diabetes may also induce structural changes in penile tissue, including smooth muscle injury and fibrosis, which further worsen erectile function. ^5^ Hormonal disturbances, including reduced testosterone levels, may further aggravate impaired libido and erectile capacity in men with diabetes. ^5^ Together, these mechanisms help explain why erectile dysfunction in men with diabetes is common, persistent, and often more severe than in men without diabetes. Erectile Dysfunction (ED) in men with DM is also associated with decreased fertility in men due to hypothalamic-pituitary-gonadal axis dysfunction, spermatogenesis and maturation disorders. ^7^

Erectile Dysfunction (ED) and reproductive disorders in men are related to testosterone level. Many studies have shown that testosterone deficiency is common in men with DM, both Type 1 DM (T1DM) and T2DM. In people with T2DM, there is a decrease in free testosterone of up to 57%, while in people with T1DM, the decrease in free testosterone reaches 20.3%. Thus, it is not an exaggeration to say that total testosterone and free testosterone levels are risk factors for DM in men. ^8^ ^,^ ^9^

Apart from testosterone as a risk factor for DM, low testosterone levels themselves are known to cause decreased sexual function and fertility in men. The low of sexual function is characterized by loss of erection, decreased sexual desire, and decreased frequency of sexual intercourse. ^10^ Research on animal model has shown that testosterone is also a determining factor in male fertility because it affects spermatogenesis. The critical processes in spermatogenesis that are influenced by testosterone are maintaining the blood testes barrier, supporting the meiosis process, the adhesion of spermatids to Sertoli cells, and the release of sperms. ^11^

Currently, there are many types of drugs commonly used to treat DM, including metformin, sulfonylureas, meglitinides, thiazolidinediones, DPP-4 inhibitors. Unfortunately, all of these drugs have side effects. Metformin, for example, causes side effects such as nausea, vomiting, abdominal bloating, diarrhea, heartburn, headache, agitation, dizziness, tiredness, chills, abdominal cramps or pain, loss of appetite, asthenia, and myalgia. ^12^ Therefore, the search for DM drugs derived from plants continues to grow. One of the medicinal plants that has the potential to have anti-diabetic properties is black pepper ( *Piper nigrum L.*). Tests on alloxan-induced diabetic rats showed that black pepper fruit extract was effective in lowering blood sugar levels and was also effective in lowering cholesterol levels. ^13^ ^,^ ^14^ The selection of black pepper ( *Piper nigrum L.*) as an intervention is supported by preclinical evidence on its major bioactive alkaloid, piperine. ^15^ In an in vitro study, piperine showed antioxidant activity by quenching free radicals and reactive oxygen species and by inhibiting lipid peroxidation, suggesting a potential to reduce oxidative tissue injury. ^16^ In an in vivo alloxan-induced diabetic mouse model, piperine significantly reduced blood glucose levels in both acute and subacute experiments, supporting its antidiabetic potential. ^15^ In another in vivo study using streptozotocin-induced diabetic rats, piperine improved diabetes-related disturbances in antioxidant defense, including glutathione-related pathways, superoxide dismutase activity, and lipid peroxidation. ^17^ Because oxidative stress is an important mechanism in diabetes-related vascular and erectile dysfunction, these in vitro and in vivo findings provide a biologically plausible basis for evaluating black pepper extract in an animal model of diabetic male sexual dysfunction. ^16^

Apart from having the potential as an anti-diabetic, black pepper fruit extract has also been proven to increase testosterone hormone levels, sexual function (libido), and spermatogenesis parameters in male rats. Mating tests on male rats given black pepper fruit extract showed a significant increase in libido, indicated by a shorter courtship latency. ^18^ The fertility parameters of male rats given black pepper fruit extract also increased, indicated by an increase in epididymal sperm concentration, spermatocyte counts, spermatid counts, and the weight of epididymis tubules. ^19^

Although black pepper has shown antidiabetic and reproductive benefits, its effect on DM-related ED is not well established. Especially, its influence on hormonal parameters, erectile function, libido parameters, spermatozoa parameters, testicular histology parameters and eNOS molecular analysis has not been evaluated. To our knowledge, this is the first to demonstrate the potential of black pepper ( *Piper nigrum L.*) fruit extract to improve sexual function as depicted in hormonal parameters, erectile function, libido parameters, spermatozoa parameters, testicular histology parameters and eNOS molecular analysis in alloxan-induced diabetic rats.

## Materials and methods

### Study design

This study was an experimental, randomized controlled laboratory to assess the effects of black pepper ( *Piper nigrum L.*) fruit extract on hormonal parameters, erectile function, libido parameters, spermatozoa parameters, testicular histology parameters and eNOS molecular analysis in alloxan-induced diabetic rats. Rats were randomly sampled into control, diabetic, extract-treated, and positive control groups to allow direct comparison of treatment outcomes. Data obtained were collected and analyzed using the IBM SPSS Statistics version 31 software (New York, USA). Normality of the data was assessed with the Shapiro-Wilk test. Since the data was considered non-parametric, Kruskal-Wallis tests were used, followed by Dunn’s post hoc test for pairwise comparisons. A *p*-value of <0.05 was considered statistically significant.

### Plant preparation

Fresh black pepper fruit is collected from a farmer in Ngarip Village, Ulubelu District, Tanggamus Regency, Lampung Province. Formal documentation regarding plant authentication, including the full name of the identifier and voucher specimen deposition in a publicly accessible herbarium, was not available and is acknowledged as a limitation of the study. Based on previous literature, *Piper Nigrum L.* is known to contain several bioactive phytoconstituents, including piperine, related amide constituents, and volatile compounds such as β-caryophyllene, limonene, α-pinene, β-pinene, δ-3-carene, sabinene, and myrcene. ^20^ We conducted a Liquid Chromatography-Mass Spectrometry (LC-MS) test to assess the phytoconstituent of black pepper extract as shown in Table 1. The fresh fruits were washed thoroughly, rinsed with clean water, and then dried. After drying, the fruit is ground into powder using a blender. The extraction was done by soaking the black pepper powder in 95% ethanol at room temperature. The supernatant collected every 24 h for three days and filtered to remove unwanted components. The filtrate was concentrated using a rotary evaporator at a temperature of 40 °C and a pressure of 60 mbar. The extract is stored in the refrigerator as stock until used.

**Figure 1.**
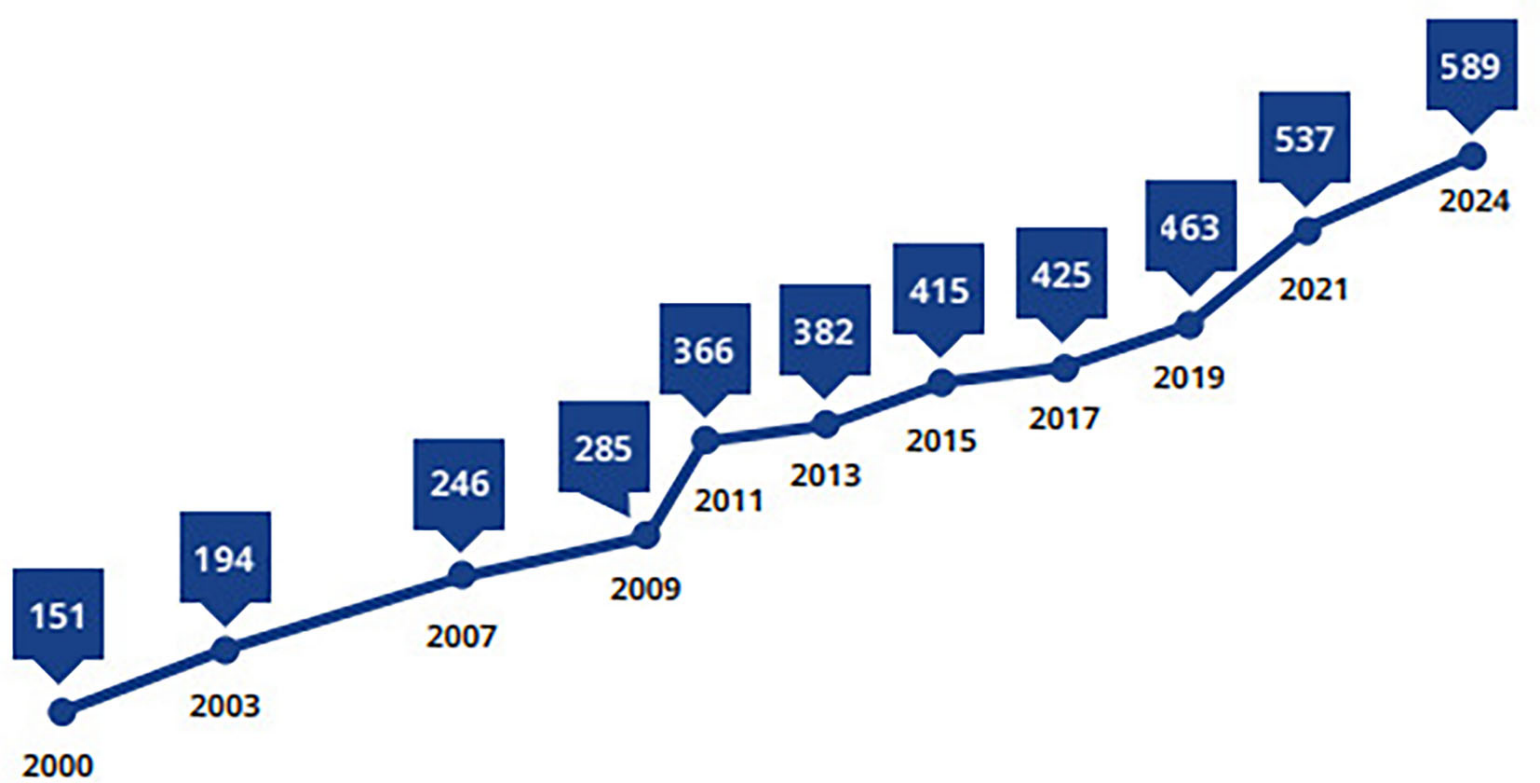
The number of people diagnosed with DM as presented in millions. ^1^

**Table 1.** The LC-MS test of black pepper extract.

No	RT	m/z	Phytochemical constituent	Molecular Formula
1	4.2	342.1698	Pipernonaline	C21H27NO3
2	4.82	356.1855	Retrofractamide B	C22H29NO3
3	6.91	314.14	(E)-Piperolein A	C19H25NO3
4	7.48	302.1383	(E,E)-Futoamide	C18H23NO3
5	8.14	274.1443	Piperamine	C16H19NO3
6	8.84	258.1495	Pinocembrine	C15H12O4
7	9.02	328.1538	Retrofractamide C	C20H27NO3
8	9.56	272.1286	Piperyline	C16H17NO3
9	10.42	286.145	Piperine	C17H19NO3
10	12.35	340.1908	Dehydropipernonaline	C21H25NO3
11	12.55	571.2779	Nigramide-F	C34H40N2O6
12	13.08	344.2221	Piperolein B	C21H29NO3
13	14.02	384.2537	Guineesine	C24H33NO3
14	14.64	396.2533	Piperchabamide C	C25H33NO3
15	14.88	452.2688	hentriacontan-16-ol	C31H64O
16	16.2	334.331	Pipyequbine	C22H39NO
17	17.14	362.3412	2,4,14-Eicosatrienoic acid isobutylamide	C24H43NO
18	17.87	374.3435	Pipereicosalidine	C25H43NO
19	18.22	364.3563	N-Isobutyl-2,4-eicosadienamide	C24H45NO
20	18.74	350.3423	1-(9-Octadecenoyl)piperidine	C23H43NO

a  = Presented in mean and Standard Deviation (SD).

Bs = Baseline fasting blood glucose.

Post = After alloxan-induced fasting blood glucose.

### Animal preparation

This study used 30 male-only *Rattus Novergicus* Sprague Dawley rats as mentioned by Federer study before, Rattus norvegicus, aged 2.5–3 months weighing 100–150 grams obtained from the animal house at the IPB University, Bogor, Indonesia. All procedures were conducted in accordance and approved with the guidelines of the Health Research Ethics Commission of the Faculty of Medicine, Lampung University (No.3468/UN26.18/PP.05.02.00/2024). The rats were acclimated for a week at a temperature of 25 °C with stable room humidity and lighting, and were given standard feed ad libitum.

The studied rats were divided into five groups of 6 individuals each. Group 1 (I) was rats that were only given standard feed. Group 2 (II) was alloxan-induced hyperglycemic rats and given feed. Groups 3 (III) and group 4 (IV) were alloxan-induced hyperglycemic rats and given black pepper extract 122.5 and 245 mg/kg BW respectively for 8 consecutive days. Group 5 (V) is alloxan-induced hyperglycemic rats given sildenafil citrate at a dose of 1 mg/kg BW at day 9, one hour before erectile function and libido assessment. Sildenafil citrate was used as an acute referencce treatment for erectile function, whwereas black pepper extract was evaluated as the test intervention over an eight-day treatment period.

Alloxan induction in studied rats was performed intraperitoneally using normal saline solvent at a dose of 150 mg/kg BW. To prevent alloxan-induced rats from suffering from severe hypoglycemia, the rats were given a 20% glucose solution (5–10 ml) orally after 6 hours. Furthermore, after being maintained for 24 hours, the rats were given further 5% glucose solution to prevent hypoglycemia. Rats are categorized to have DM if they experience glycosuria and hyperglycemia with blood glucose levels of 200 to 300 mg/dL. ^21^ In this study, alloxan induction was used to establish an insulin-deficient, type 1-like diabetic model, as alloxan selectively damages pancreatic β-cells. ^22^ The level of fasting blood glucose on each rat before and after alloxan-induced hyperglycemic state was shown in Table 2.

**Table 2.** Fasting blood glucose levels of the rats before and after alloxan-induced.

Animals	Fasting blood glucose (mg/dL)								
	Group I (only fed)	Group II (alloxan)		Group III (extract 122.5 mg/kg BW)		Group IV (extract 245 mg/kg BW)		Group V (sildenafil citrate)	
	Bs	Bs	Post	Bs	Post	Bs	Post	Bs	Post
1	167	134	247	156	254	179	247	182	247
2	147	152	231	173	501	146	230	177	254
3	186	132	230	164	501	158	501	167	231
4	172	145	210	133	206	189	254	168	281
5	144	166	220	152	231	143	247	152	500
6	127	156	206	162	230	182	501	173	519
Mean ± SD	157.17 ± 21.61 ^a^	147.5 ± 13.14 ^a^	224 ± 15.17 ^a^	156.67 ± 13.65 ^a^	320.5 ± 140.64 ^a^	166.17 ± 19.73 ^a^	330 ± 132.69 ^a^	169.83 ± 10.38 ^a^	338.67 ± 133.44 ^a^

^a^ Presented in mean and Standard Deviation (SD).

Bs = Baseline fasting blood glucose.

Post = After alloxan-induced fasting blood glucose.

Phosphodiesterase-5 inhibitor (PDE-5i) therapy was given one hour before observation of erectile function and libido assessment. The therapy was done using Sildenafil Citrate, a drug from the PDE-5i at a dose of 1 mg/kg BW as positive control for recovery of ED caused by DM. ^23^ Sildenafil treatment was done following Gurbuz et al. (2015) to decrease advanced glycation end products, Malondialdehyde (MDA) and Inducible Nitric Oxide Synthase (iNOS), to preserve Neuronal Nitric Oxide Synthase (nNOS) and Cyclic Guanosine Monophosphate (cGMP) contents in penile tissue. ^24^

At the end of the experimental period, following completion of all functional assessments, the rats were euthanized by injection of ketamine at a dose of 100 mg/kg BW and cervical dislocation in accordance with the approved institutional ethics protocol.

### Hormonal assessment

In this study, Intratesticular Testosterone (ITT) levels were measured from testicular tissue homogenates using a commercial Enzyme-Linked Immunosorbent Assay (ELISA) kit in μg. Excised testes were mechanically homogenized, and the homogenates were centrifuged at 10,000x g for 10 min at 4 °C. The resulting supernatants were collected and stored at 2–8 °C until analysis. Testosterone concentrations in the supernatants were quantified using a testosterone ELISA kit (R&D Systems; catalog no. KGE010; Minneapolis, MN, USA) according to the manufacturer’s instructions. Samples were diluted 1:10 prior to assay (50 μL sample + 450 μL Calibrator Diluent RD5–48). Wash buffer was prepared by diluting the Wash Buffer Concentrate. The substrate solution was prepared by mixing Color Reagents A and B at equal volumes within 15 min before use and protected from light (approximately 200 μL per well). Testosterone standards were reconstituted with distilled water to obtain a 100 ng/mL stock solution, mixed thoroughly, and allowed to stand for at least 15 minutes before preparation of the standard curve. The assay range was 0–10 ng/mL with a stated sensitivity of 0.041 ng/mL.

### Erectile function assessment

Erectile function of the rats was assessed on the ninth day one hour before the mating test was performed. The animals were placed in supine position with partial restraint. The preputial sheath was pushed behind the glans penis and held in this manner for a period of 15 min to elicit penile reflex. Total Penile Reflexes (TPR) for each animal were calculated as the sum of Erection (E), Long Flips (LF) and Quick Flips (QF) frequencies (n). Mathematically, it can be expressed using the following formula TPR = E + QF + LF. ^25^ ^,^ ^26^

### Libido assessment

To determine the libido level of the studied rats, a mating test was conducted on the ninth day. The mating test was conducted by placing male and female rats in a cage with a partition in the middle. The cage was placed in a room with low lighting. Observation and analysis of the mating behavior of rats were conducted through video recording. There were three mating behavior parameters assessed, namely courtship latency, mounting latency, and mounting frequency Courtship Latency (CL), Mounting Latency (ML), and Mounting Frequency (MF). The CL is the time in seconds (s) from opening of the partition until the male initiated investigatory or courtship behavior toward the female, including anogenital sniffing or contact with the female’s body. The ML is the time in seconds (s) from the partition being opened until the male mounts the female’s back. The MF is the number of times the male mounts the female’s back during the 30 minutes (1800 seconds) of the mating test.

### Sperm analysis and testis histology examination

After the erectile function and mating behavior were assessed, sperm and histological analysis of the studied rats were performed. The sperm analysis and testis histology examination were performed in Anatomy Pathology Laboratorium of Faculty of Medicine, Lampung University. The rats were terminated by injection of ketamine at a dose of 100 mg/kg BW and cervical dislocation. A total of 30 right testes and cauda epididymis were removed using a dissecting kit. The cauda epididymis were pinched to squeeze the spermatozoa into the petri dish. Subsequently, a suspension of spermatozoa were formed after being mixed with NaCL 0.9%, as mentioned in previous study. ^27^ Spermatozoa were counted (Sperm Concentration [SC]) using a Neubauer’s hemocytometer under a light microscope (400x) and expressed as millions/ml. Sperm motility was evaluated by counting motile and immotile sperms. Sperm morphology was assessed from a smear of the epididymal filtrate prepared on a clean glass slide with 1% eosin staining. After the object dried, observation was done under Richter Optica HS-3B-3’s light microscope (China) at 400x magnification and abnormalities of either head or tail were noted. Spermatozoa parameters were only assessed in studied rats of group 1 to group 4. Testicular histology examination was performed using Haematoxylin & Eosin (HE) staining and spermatocyte were counted in 10 random seminiferous tubule cross-sections per testis, and Leydig cells were counted in 10 non-overlapping interstitial fields with thickness of 4–5 μm; counts were normalized to area using National Institute of Health ImageJ software version 1.8.0 (New York, USA) (calibrated to μm ^2^) at 400x magnification. ^28^

### Molecular analysis

Molecular analysis was performed with the assessment of Endothelial Nitric Oxide (eNOS) mRNA expression in corpus cavernosum. Corpus cavernosum tissues were collected immediately after euthanasia and processed under RNase-free conditions. Approximately 30 mg of corpus cavernosum was excised rapidly, snap-frozen in liquid nitrogen, and stored at −80 °C until analysis. Total RNA was extracted using the RNeasy Mini Kit (Qiagen, Hilden, Germany) according to the manufacturer’s instructions. Briefly, frozen tissue was mechanically homogenized in liquid nitrogen and lysed in 600 μL Buffer RLT Plus, then homogenized by passage through a 20-gauge needle using an RNase-free syringe. Lysates were centrifuged (10.000 rpm for 3 minutes) and the supernatant was applied to a gDNA Eliminator spin column. The flow-through was mixed with 70% ethanol and loaded onto an RNeasy spin column, followed by sequential washes with Buffer RW1 and Buffer RPE. RNA was eluted in 30–50 μL RNase-free water. RNA concentration and purity were assessed using a sub-microliter UV-Vis spectrophotometer (NanoPhotometer; Implen, Munich, Germany) by A260/A280 and A260/A230 ratios, and RNA integrity was evaluated by agarose gel electrophoresis with UV visualization. To minimize genomic DNA contamination, RNA was treated with DNase prior to reverse transcription. Complementary DNA (cDNA) was synthesized using the iScript™ cDNA Synthesis Kit (Bio-Rad, California, USA) following the manufacturer’s protocol. Relative eNOS mRNA expression was quantified by Real-Time Quantitative Polymerase Chain Reaction (RT-qPCR) using LineGene Mini S (Bioer, Hangzhou, China) with RealMOD™ Green W2 2x qPCR mix (iNtRON, Seongnam-si, Republic of Korea). Primer sequences were: eNOS forward 5′-CTGCTGCCCCAGATATCTTC-3′ and reverse 5′-CAGGTACTGCAGTCCCTCCT-3′; β-actin (reference gene) forward 5′-CAACTCCCTCAAGATTGTCAGCAA-3′ and reverse 5′-GGCATGGACTGTGGTCATGA-3′. Each reaction contained 2 μL cDNA, 10 μL 2x qPCR mix, 0.5 μL forward primer, 0.5 μL reverse primer, and nuclease-free water to the final reaction volume. Thermal cycling was performed for 35 cycles with denaturation at 94 °C for 30 s, annealing at 54 °C (eNOS) or 55 °C (β-actin) for 1 min, and extension at 72 °C. A no-template control was included to monitor contamination. Ct values were normalized to β-actin and relative expression was calculated using the 2^-ΔΔCt (Livak) method, with the diabetic control (alloxan) group used as the calibrator.

## Results

### Hormonal

Hormonal parameters as presented by ITT concentrations of the studied rats are presented in Table 3. The alloxan-induced group (group II) showed a lower ITT level (5.6 ± 1.32 μg) compared with the normal control (group I: 8.9 ± 2.04 μg; p = 0.003). The black pepper extract 122.5 mg/kg BW group (group III) demonstrated a similarly low ITT level (5.6 ± 1.38 μg) and remained significantly lower than group I (p = 0.004), but was not significantly different from group II (p = 0.874). In contrast, the black pepper extract 245 mg/kg BW group (group IV) had a higher ITT level (8.8 ± 2.00 μg) and was significantly higher than group II (p = 0.003) and group III (p = 0.004), while showing no difference compared with group I (p = 0.984). The sildenafil citrate group (group V) recorded the highest ITT level (9.7 ± 0.76 μg) and was significantly higher than group II (p < 0.001) and group III (p = 0.001), with no significant differences compared with group I (p = 0.389) or group IV (p = 0.378).

**Table 3.** Hormonal Parameters of Studied Rats.

Group	ITT (μg)	*p*-value ^#^
I (only fed)	8.9 ± 2.04 ^a^	vs II = **0.003**; vs III = **0.004**; vs IV = 0.984; vs V = 0.389
II (alloxan)	5.6 ± 1.32 ^a^	vs I = **0.003**; vs III = 0.874; vs IV = **0.003**; vs V = **<0.001**
III (extract 122.5 mg/kg BW)	5.6 ± 1.38 ^a^	vs I = **0.004**; vs II = 0.874; vs IV = **0.004**; vs V = **0.001**
IV (extract 245 mg/kg BW)	8.8 ± 2.00 ^a^	vs I = 0.984; vs II = **0.003**; vs III = **0.004**; vs V = 0.378
V (sildenafil citrate)	9.7 ± 0.76 ^a^	vs I = 0.389; vs II = **<0.001**; vs III = **0.001**; vs IV = 0.378

^a^ Presented in mean and Standard Deviation (SD)

^#^ Dunn’s Post Hoc test.

### Erectile function

In the evaluation of erectile function, the control group (I) showed mean values of 3.67 ± 0.82 for quick flip (QF), 1.33 ± 0.52 for long flip (LF), 4.67 ± 0.82 for erection (ER), and 9.67 ± 1.03 for total penile reflexes (TPR), as presented in Table 4. The alloxan-induced group (II) showed lower mean values for QF (1.83 ± 0.75), LF (0.33 ± 0.52), ER (3.83 ± 0.75), and TPR (6.00 ± 1.26). Based on post hoc analysis of TPR, group I differed significantly from group II (p < 0.001), but not from group III (9.33 ± 1.03; p = 0.246), group IV (6.83 ± 0.98; p = 1.000), or group V (8.17 ± 1.17; p = 0.135). Group II had a significantly lower TPR than group III (p = 0.020), group IV (p < 0.001), and group V (p = 0.038). No significant differences were found between group III and group IV (p = 0.747), group III and group V (p = 1.000), or group IV and group V (p = 0.436). Overall, the lowest TPR was observed in the diabetic group, whereas the control and low-dose extract groups showed the highest TPR values.

**Table 4.** Erectile function parameters of studied rats.

Group	QF (n)	LF (n)	ER (n)	TPR (n)	TPR *p*-value ^#^
I (only fed)	3,67 ± 0,82 ^a^	1.33 ± 0.52 ^a^	4.67 ± 0.82 ^a^	9.67 ± 1.03 ^a^	vs II = **<0.001**; vs III = 0.246; vs IV = 1.000; vs V = 0.135
II (alloxan)	1.83 ± 0.75 ^a^	0.33 ± 0.52 ^a^	3.83 ± 0.75 ^a^	6.00 ± 1.26 ^a^	vs I = **<0.001**; vs III = **0.02**; vs IV = < **0.001**; vs V = **0.038**
III (extract 122.5 mg/kg BW)	3.33 ± 1.03 ^a^	1.67 ± 0.52 ^a^	4.33 ± 0.52 ^a^	9.33 ± 1.03 ^a^	vs I = 0.246; vs II = **0.02**; vs IV = 0.747; vs V = 1.000
IV (extract 245 mg/kg BW)	2.83 ± 0.75 ^a^	0.67 ± 0.82 ^a^	3.33 ± 0.82 ^a^	6.83 ± 0.98 ^a^	vs I = 1.000; vs II = **<0.001**; vs III = 0.747; vs V = 0.436
V (sildenafil citrate)	2.17 ± 0.75 ^a^	1.33 ± 0.52 ^a^	4.67 ± 0.52 ^a^	8.17 ± 1.17 ^a^	vs I = 0.135; vs II = **0.038**; vs III = 1.000; vs IV = 0.436

^a^ Presented in mean and Standard Deviation (SD).

^#^ Dunn’s Post Hoc test.

n = Frequency.

### Libido

The effect of giving black pepper extract on the libido of rats using the parameters of CL, ML and MF can be seen in Table 5. Based on the data in Table 5, it can be seen that the administration of black pepper fruit extract significantly improved the decreased libido due to hyperglycemia caused by alloxan induction. Based on the values of the CL, ML and MF, it was revealed that the best dose of black pepper extract to restore the libido of hyperglycemic male rats was a dose of 122.5 mg/kg BW. The therapeutic effect with sildenafil citrate also significantly improved the libido of rats.

**Table 5.** Libido parameters of Courtship Latency (CL), and Mount Latency (ML) of studied rats.

Group	CL (s)	CL *p*-value ^^^	ML (s)	ML *p*-value ^#^
I (only fed)	5.17 ± 0.41 ^a^	vs II = **0.003**; vs III = 0.060; vs IV = 0.241; vs V = **0.005**	8.50 ± 3.51 ^a^	vs II = **<0.001**; vs III = **0.033**; vs IV = **0.006**; vs V = **<0.001**
II (alloxan)	21.00 ± 9.47 ^a^	vs I = **0.003**; vs III = **0.013**; vs IV = **0.003**; vs P2 = **0.003**	37.17 ± 6.31 ^a^	vs I = **<0.001**; vs III = **0.009**; vs IV = **<0.001**; vs V = 0.075
III (extract 122.5 mg/kg BW)	5.50 ± 0.55 ^a^	vs I = 0.06; vs II = **0.013**; vs IV = 0.204; vs V = 1.000	19.00 ± 10.81 ^a^	vs I = **0.033**; vs II = **0.009**; vs IV = 0.464; vs V = **0.007**
IV (extract 245 mg/kg BW)	7.00 ± 1.10 ^a^	vs I = 0.241; vs II = **0.003**; vs III = 0.204; vs V = **0.019**	27.67 ± 6.53 ^a^	vs I = **0.006**; vs II = **<0.001**; vs III = 0.464; vs V = **0.026**
V (sildenafil citrate)	9.00 ± 4.82 ^a^	vs I = **0.005**; vs II = **0.003**; vs III = 1.000; vs IV = **0.019**	16.00 ± 8.27 ^a^	vs I = **<0.001**; vs II = 0.075; vs III = **0.007**; vs IV = **0.026**

^a^ Presented in mean and Standard Deviation (SD).

^^^ Mann-Whitney test.

^#^ Dunn’s Post Hoc test.

s = Seconds.

Courtship Latency (CL) and ML showed significant differences among groups as shown in Table 5 and Table 6. The alloxan group (II) had the longest CL (21.00 ± 9.47, p = 0.003 vs group I) and ML (37.17 ± 6.31, p < 0.001 vs group I). In contrast, the extract 122.5 mg/kg BW group (III) demonstrated CL (5.50 ± 0.55) and ML (19.00 ± 10.81), both significantly different from group II (p = 0.013 and p = 0.009, respectively) and closer to group I. The extract 245 mg/kg BW group (IV) also showed shorter CL (7.00 ± 1.10) and ML (27.67 ± 6.53) compared with group II (p = 0.003 and p < 0.001, respectively). The sildenafil group (V) exhibited CL (9.00 ± 4.82) and ML (16.00 ± 8.27), with significant differences from group II (p = 0.003 and p = 0.075, respectively).

**Table 6.** Mount Frequency (MF) of studied rats.

Group	MF (n/30 mins)	MF *p*-value ^#^
I (only fed)	17.00 ± 3.74 ^a^	vs II = **<0.001**; vs III = 0.351; vs IV = 0.649; vs V = 0.157
II (alloxan)	7.17 ± 1.83 ^a^	vs I = **<0.001**; vs III = **0.002**; vs IV = **<0.001**; vs V = **0.006**
III (extract 122.5 mg/kg BW)	18.05 ± 5.99 ^a^	vs I = 0.351; vs II = **0.002**; vs IV = 0.17; vs V = 0.615
IV (extract 245 mg/kg BW)	13.33 ± 3.45 ^a^	vs I = 0.649; vs II = **<0.001**; vs III = 0.17; vs IV = 0.066
V (sildenafil citrate)	14.83 ± 5.74 ^a^	vs I = 0.157; vs II = **0.006**; vs III = 0.615; vs IV = 0.066

^a^ Presented in mean and Standard Deviation (SD).

^#^ Dunn’s Post Hoc test.

n/30 mins = Frequency in 30 minutes.

Mount Frequency (MF) was lowest in group II (7.17 ± 1.83), significantly different from group I (17.00 ± 3.74, p < 0.001). Group III (18.05 ± 5.99) was not significantly different from group I (p = 0.351), while group IV (13.33 ± 3.45) and group V (14.83 ± 5.74) showed higher MF compared with group II (p < 0.001 and p = 0.006, respectively).

### Sperm analysis

In sperm concentration (SC), the control group (I) recorded a mean of 75.81 ± 52.9 millions/mL, which was significantly higher than group II (12.6 ± 1.3; p = 0.002) and group III (19.2 ± 6.7; p = 0.004), but not different from group IV (62.95 ± 29.4; p = 0.483) as shown in Table 7. Compared with group V (158.16 ± 29.8), group I was significantly lower (p < 0.001). Group II had significantly lower SC compared with group IV (p = 0.01) and group V (p < 0.001), but no significant difference from group III (p = 0.877). Group III showed significantly lower SC than group IV (p = 0.019) and group V (p < 0.001). Group IV exhibited significantly lower SC than group V (p < 0.001). Overall, group V demonstrated the highest SC among all groups with significant differences in all comparisons (p < 0.001).

**Table 7.** Sperm analysis parameters of sperm concentration, and sperm progressive motility of studied rats.

Group	SC (millions/mL)	SC *p*-value ^#^	SPM	SPM *p*-value ^#^
I (only fed)	75.81 ± 52.9 ^b^	vs II = **0.002**; vs III = 0.004*; vs IV = 0.483; vs V = **<0.001**	57 ± 33 ^a^	vs II = **0.024**; vs III = 0.07; vs IV = 0.121; vs V = 0.556
II (alloxan)	12.6 ± 1.3 ^a^	vs I = **0.002**; vs III = 0.877; vs IV= **0.01**; vs V = **<0.001**	27 ± 30 ^a^	vs I = **0.024**; vs III = 0.697; vs IV = 0.436; vs V = **0.006**
III (extract 122.5 mg/kg BW)	19.2 ± 6.7 ^a^	vs I = **0.004**; vs II = 0.877; vs IV = **0.019**; vs V = **<0.001**	31.8 ± 23 ^a^	vs I = 0.07; vs II = 0.697; vs IV = 0.721; vs V = **0.021**
IV (extract 245 mg/kg BW)	62.95 ± 29.4 ^a^	vs I = 0.483; vs II = **0.01**; vs III = **0.019**; vs V = < **0.001**	36.6 ± 23 ^a^	vs I = 0.121; vs II = 0.436; vs III = 0.721; vs V = **0.037**
V (sildenafil citrate)	158.16 ± 29.8 ^a^	vs I = **<0.001**; vs II = **<0.001**; vs III = **<0.001**; vs IV = **<0.001**	65 ± 35 ^a^	vs I = 0.556; vs II = 0.006*; vs III = **0.021**; vs IV = **0.037**

^a^ Presented in mean and Standard Deviation (SD).

^#^ Dunn’s Post Hoc test.

For SPM as shown in Table 7, the control group (I) recorded 57 ± 33, which was significantly higher than group II (27 ± 30; p = 0.024), but not different from group III (31.8 ± 23; p = 0.07) or group IV (36.6 ± 23; p = 0.121). Compared with group V (65 ± 35), no significant difference was observed (p = 0.556). Group II showed significantly lower SPM than group V (p = 0.006) but not significantly different from group III (p = 0.697) or group IV (p = 0.436). Group III had significantly lower SPM compared with group V (p = 0.021) but did not differ significantly from group IV (p = 0.721). Group IV also showed significantly lower SPM than group V (p = 0.037). Thus, group V demonstrated the highest SPM, with significant superiority over groups II–IV.

Regarding SNM as presented in Table 8, the control group (I) recorded 75 ± 13.7, which was significantly higher than group II (35 ± 10.8; p < 0.001) and group III (40.9 ± 7.8; p < 0.001), but not different from group IV (62 ± 14.7; p = 0.119) or group V (82.9 ± 5.7; p = 0.273). Group II had significantly lower SNM than all other groups (p ≤ 0.04). Group III also showed significantly lower SNM compared with group IV (p = 0.017) and group V (p < 0.001). Group IV exhibited significantly lower SNM than group V (p = 0.011). Among all groups, group V demonstrated the highest SNM, with significant differences against groups II–IV.

**Table 8.** Sperm analysis parameters of sperm normal morphology of studied rats.

Group	SNM	SNM *p*-value ^#^
I (only fed)	75 ± 13.7 ^a^	vs II = **<0.001**; vs III = **<0.001**; vs IV = 0.119; vs V = 0.273
II (alloxan)	35 ± 10.8 ^a^	vs I = **<0.001**; vs III = **0.04**; vs IV = **<0.001**; vs V = < **0.001**
III (extract 122.5 mg/kg BW)	40.9 ± 7.8 ^a^	vs I = **<0.001**; vs II = **0.04**; vs IV = **0.017**; vs V = **<0.001**
IV (extract 245 mg/kg BW)	62 ± 14.7 ^a^	vs I = 0.119; vs II = **<0.001**; vs III = **0.017**; vs V = **0.011**
V (sildenafil citrate)	82.9 ± 5.7 ^a^	vs I = 0.273; vs II = **<0.001**; vs III = **<0.001**; vs IV = **0.011**

^a^ Presented in mean and Standard Deviation (SD).

^#^ Dunn’s Post Hoc test.

### Testicular histology analysis

Table 9 presents the examination results of the testicular histology of alloxan-induced diabetic rats treated with black pepper extract. The data in the table shows that black pepper extract at a dose of 122.5 mg/kg BW significantly restored the number of LC and StC. As shown in Figure 2, group III with black pepper extract of 122.5 mg/kg BW showed a higher amounts of StC as compared with group II without black pepper extraction.

**Table 9.** Testicular histology parameters.

Group	LC	LC *p*-value	StC	StC *p*-value
I (only fed)	60.83 ± 5.1 ^a^	vs II = **<0.001**; vs III = **<0.001**; vs IV = 0.581; vs V = **0.001**	511 ± 73.4 ^a^	vs II = 0.593; vs III = **<0.001**; vs IV = 0.294; vs V = **0.013**
II (alloxan)	30.50 ± 3.86 ^a^	vs I = **<0.001**; vs III = **0.035**; vs IV = **<0.001**; vs V = **0.001**	491 ± 37.0 ^a^	vs I = 0.593; vs III = **<0.001**; vs IV = 0.119; vs V = **0.042**
III (extract 122.5 mg/kg BW)	59.33 ± 4.0 ^a^	vs I = **<0.001**; vs II = **0.035**; vs IV = **<0.001**; vs V = **<0.001**	319.4 ± 64.59 ^a^	vs I = **<0.001**; vs II = **<0.001**; vs IV = **<0.001**; vs V = **0.014**
IV (extract 245 mg/kg BW)	50.67 ± 2.73 ^a^	vs I = 0.581; vs II = **<0.001**; vs III = **<0.001**; vs IV = **0.003**	640 ± 86.5 ^a^	vs I = 0.294; vs II = 0.119; vs III = **<0.001**; vs V = **0.001**
V (sildenafil citrate)	32.60 ± 3.28 ^a^	vs I = **0.001**; vs II = **<0.001**; vs III = **<0.001**; vs P1 = **0.003**	414 ± 32.5 ^a^	vs I = **0.013**; vs II = **0.042**; vs III = **0.014**; vs IV = **0.001**

^a^ Presented in mean and Standard Deviation (SD).

# Dunn’s Post Hoc test.

**Figure 2.**
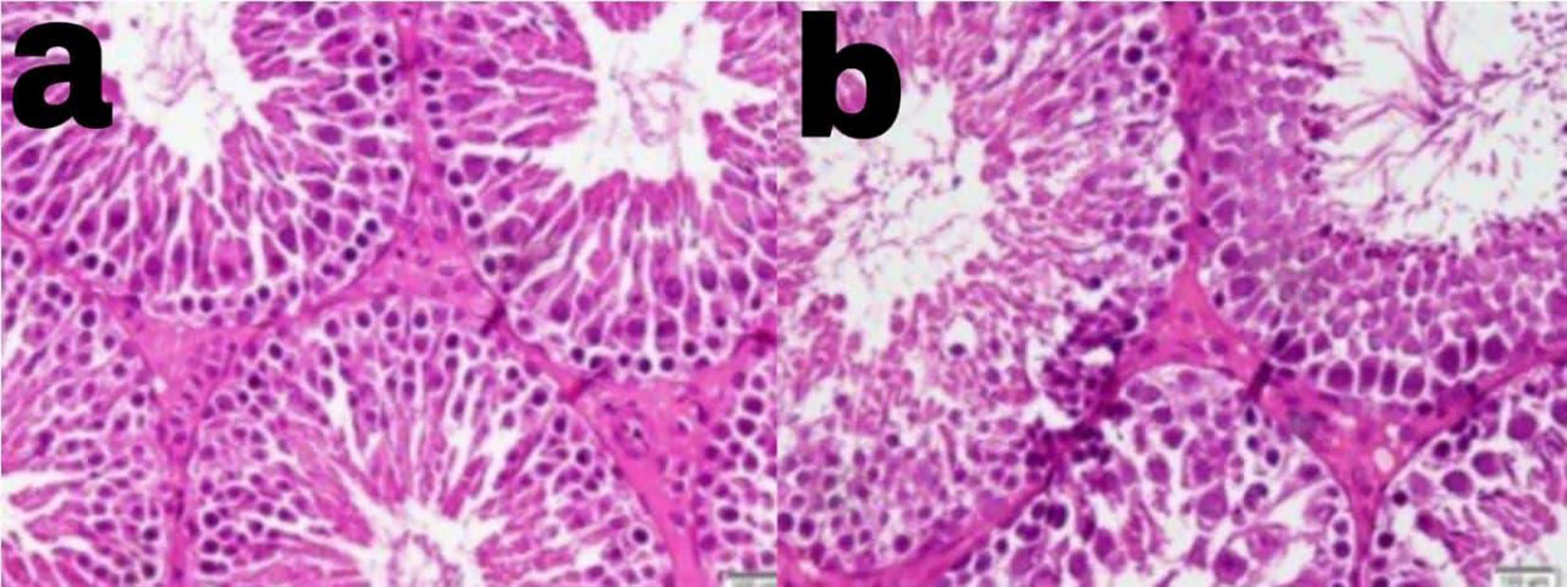
Higher amounts of spermatocyte was observed in group III with black pepper extract 122.5 mg/kg BW (a) as compared in group II without black pepper extract (b).

### Molecular analysis

The eNOS mRNA expression in the corpus cavernosum was assessed by RT-qPCR as shown in Table 10. The alloxan group (group II) was used as the calibrator (fold change = 1.00). Relative eNOS expression was upregulated in all treatment groups compared with Group II. The black pepper extract 122.5 mg/kg BW group (group III) showed the highest eNOS expression (36718403.59), followed by the sildenafil citrate group (group V) (12,677.65) and the black pepper extract 245 mg/kg BW group (group IV) (167.73). Overall, eNOS gene expression differed significantly among groups (p < 0.001).

**Table 10.** Molecular Analysis of eNOS mRNA Expression on Studied Rats.

Group	eNOS mRNA Expression
II (alloxan)	1.00
III (extract 122.5 mg/kg BW)	36718403.59
IV (extract 245 mg/kg BW)	167.73
V (sildenafil citrate)	12677.65

## Discussion

**Figure 3.**
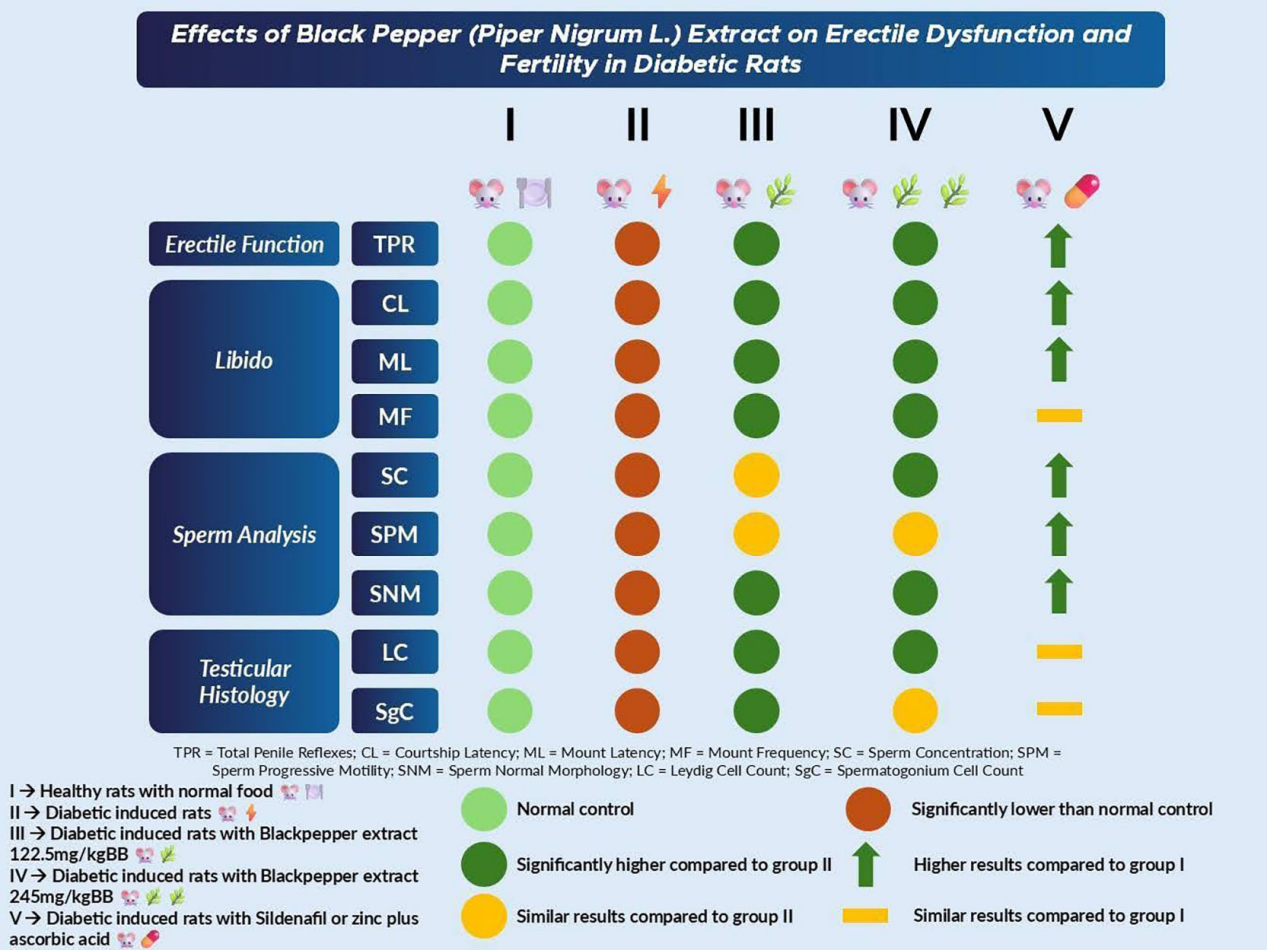
Summary of results.

In the present study, black pepper ( *Piper nigrum L.*) fruit extract produced selective, dose-dependent improvements in sexual and reproductive outcomes in alloxan-induced diabetic rats. Diabetes-related male sexual and reproductive dysfunction is biologically plausible because diabetes can impair the hypothalamic–pituitary–gonadal axis, damage Leydig and germ cells, worsen semen quality, and promote erectile dysfunction through combined endocrine, vascular, neural, and oxidative-stress mechanisms. ^29^ The diabetic model used here should also be interpreted as an insulin-deficient, type 1-like state, because alloxan preferentially accumulates in pancreatic β-cells and induces reactive oxygen species-mediated β-cell injury. ^30^ Against this background, the partial recovery observed after black pepper extract suggests that its effects may not be mediated by a single pathway, but rather by a combination of antioxidant, endothelial, and testicular protective mechanisms. ^29^ This interpretation is supported by recent preclinical evidence showing that piperine, the principal alkaloid of Piper nigrum, can improve erectile function in diabetic rats while reducing oxidative stress, apoptosis, and cavernosal fibrosis. ^31^ Accordingly, the findings of the present study are best interpreted as early experimental effects in a short-term diabetic model, rather than definitive reversal of chronic diabetic sexual dysfunction. In Figure 3, we presented the summary of our results based on erectile function, libido, sperm analysis, and testicular histology.

In the present study, alloxan-induced diabetes was associated with impaired erectile and libido-related behavior, as reflected by lower TPR, prolonged courtship and mount latency, and reduced mount frequency compared with the normal control group. Diabetic erectile dysfunction is biologically plausible because it is a multifactorial disorder involving endothelial dysfunction, reduced nitric oxide bioavailability, oxidative stress, autonomic and peripheral neuropathy, hormonal disturbance, and structural alterations in penile tissue. ^5^ In male rat behavioral models, longer mount latency and lower mount frequency generally indicate reduced sexual motivation and poorer copulatory performance, whereas shorter latency and higher mounting activity are consistent with improved sexual drive and sexual performance. ^32^ Against this background, the 122.5 mg/kg BW black pepper group showed the clearest overall recovery of erectile/libido-related outcomes, with TPR values approaching those of the normal control and favorable courtship latency, mount latency, and mount frequency results, whereas the 245 mg/kg BW extract group and sildenafil group also improved several libido-related measures relative to the diabetic group. The fact that behavioral and erectile endpoints did not improve identically across all treatment groups suggests that early recovery was not mediated by a single pathway, but may reflect a combination of vascular, endothelial, neural, and behavioral mechanisms rather than testosterone alone. This interpretation is supported by recent preclinical evidence showing that piperine, the principal alkaloid of Piper nigrum, improved erectile function in diabetic rats while reducing oxidative stress and apoptosis in corpus cavernosum tissue. ^31^

With respect to the endocrine and semen-related outcomes, alloxan induction was associated with lower ITT, sperm concentration, sperm progressive motility, and sperm normal morphology compared with the normal control group. In the treatment groups, the 245 mg/kg BW black pepper extract dose restored ITT to a level comparable to the normal control, whereas the 122.5 mg/kg BW dose remained similar to the diabetic group. A similar pattern was observed for sperm concentration and sperm morphology: the higher-dose extract showed clearer recovery than the lower-dose extract, while sildenafil produced the highest sperm concentration and the best sperm progressive motility. By contrast, the lower-dose extract did not significantly improve sperm concentration or progressive motility relative to the diabetic group, suggesting that the effect of black pepper extract on semen quality was endpoint-specific and dose-dependent within the short treatment period. This overall pattern is biologically plausible because diabetes is known to impair male reproductive function through oxidative stress, endocrine dysregulation, testicular cell injury, and disruption of spermatogenesis, which together contribute to lower testosterone production and poorer semen quality. ^29^ In addition, advanced glycation end products have been shown to suppress testosterone secretion by rat Leydig cells through oxidative stress and endoplasmic reticulum stress, providing a mechanistic explanation for the low ITT seen in the diabetic group. ^33^ The more favorable testosterone response at 245 mg/kg BW may therefore indicate better preservation of Leydig-cell steroidogenic function, whereas the modest effect on sperm progressive motility suggests that recovery of sperm movement may require additional improvement in epididymal, mitochondrial, or membrane-related function, which may not occur as rapidly as endocrine recovery. ^29^ This interpretation is also consistent with previous work showing that piperine can stimulate Leydig-cell development and steroidogenic protein expression, while other studies have reported that prolonged or higher exposure to piperine may have divergent or even adverse effects on spermatogenesis, indicating that its reproductive actions are complex and may depend on dose, duration, and endpoint assessed. ^34^

Histologically, the diabetic group showed a marked reduction in Leydig cell count compared with the normal control, whereas the 122.5 mg/kg BW black pepper extract group showed the clearest recovery, the 245 mg/kg BW group showed partial recovery, and the sildenafil group remained close to the diabetic group; by contrast, the spermatocyte-count pattern was less linear across groups ( Table 7). Leydig cells are the principal androgen-producing cells of the testis, and their functional integrity is essential for testosterone synthesis and the maintenance of male reproductive capacity. ^34^ Diabetic testicular dysfunction is known to involve structural testicular injury, impaired spermatogenesis, altered testosterone secretion, and increased apoptosis or dysfunction of Leydig cells under hyperglycemic and oxidative-stress conditions. ^35^ Against this background, the recovery of Leydig cell count in the extract-treated groups suggests that black pepper extract may exert at least partial testicular protective effects in diabetic animals. ^29^ ^,^ ^35^ This interpretation is biologically plausible because piperine, the principal alkaloid of *Piper nigrum* L., has been reported to increase Leydig cell number, promote Leydig-cell maturation, and upregulate multiple steroidogenic proteins in rat testes. ^34^ However, the spermatocyte-count results in the present study should be interpreted more cautiously, because the pattern across groups was not fully concordant with the Leydig-cell data or with the expected direction of diabetic testicular injury ( Table 7). Such divergence is still biologically possible, as piperine has also been reported to show diverged effects in the testis by stimulating Leydig-cell development while at the same time inhibiting spermatogenesis. ^34^ Therefore, the histological findings of the present study suggest that black pepper extract may provide stronger support for Leydig-cell preservation than for uniform restoration of all spermatogenic cell populations, and longer-duration studies with more detailed seminiferous-tubule analysis are needed to clarify these effects. ^35^

All treatment groups showed higher relative eNOS mRNA expression than the diabetic control group, with the largest increase observed in the 122.5 mg/kg BW black pepper extract group ( Table 8). Because eNOS is a key source of nitric oxide in the corpus cavernosum and contributes importantly to penile smooth-muscle relaxation and maintenance of erection, this directional increase is biologically consistent with the improvement seen in erectile-related outcomes. ^36^ Diabetic erectile dysfunction is strongly associated with endothelial dysfunction, and experimental diabetic models have shown impaired eNOS signaling in penile tissue. ^5^ In a diabetic rat model, Musicki et al. demonstrated that phosphorylated eNOS in the penis becomes inactivated through O-GlcNAc modification at Ser-1177, directly linking hyperglycemia to defective eNOS-mediated erectile signaling. ^37^ Similarly, Bivalacqua et al. showed that RhoA/Rho-kinase activity suppresses eNOS in the diabetic corpus cavernosum, further supporting the role of endothelial nitric oxide deficiency in diabetes-associated erectile dysfunction. ^38^ Against this mechanistic background, the upward shift in eNOS expression after black pepper treatment may indicate partial restoration of endothelial NO signaling in the diabetic penis. ^36^ This interpretation is also compatible with recent experimental evidence showing that piperine improved diabetic erectile dysfunction in rats while reducing oxidative stress and apoptosis through PI3K/AKT/NRF2-related signaling. ^31^ Hyperglycemia-induced oxidative stress is highly relevant to diabetic erectile dysfunction because excessive reactive oxygen species reduce nitric oxide bioavailability and promote endothelial dysfunction in penile tissue. Oxidative stress may also contribute to eNOS uncoupling or impaired eNOS activation, thereby weakening NO-mediated smooth-muscle relaxation in the corpus cavernosum. ^39^ ^,^ ^40^ Therefore, the directional increase in eNOS expression observed after black pepper treatment may be mechanistically relevant not only as a molecular change, but also as a possible indicator of partial restoration of the oxidative stress-NO signaling axis in diabetic erectile dysfunction. ^39^ ^,^ ^40^

The overall pattern of the present study suggests that the effects of black pepper fruit extract were dose-dependent and endpoint-specific, rather than uniformly beneficial across all sexual and reproductive outcomes. Specifically, the 122.5 mg/kg BW dose appeared to show more favorable recovery in several erectile and libido-related parameters, whereas the 245 mg/kg BW dose showed clearer improvement in intratesticular testosterone, sperm concentration, and sperm normal morphology. This non-uniform pattern is biologically plausible because black pepper is a chemically complex extract, and its activity cannot be attributed to a single mechanism alone. Piperine is the principal bioactive alkaloid of black pepper, but Piper nigrum also contains multiple volatile constituents such as β-caryophyllene, limonene, α-pinene, β-pinene, sabinene, δ-3-carene, and myrcene, which may contribute to heterogeneous biological effects. ^41^ In addition, previous experimental work suggests that piperine itself may exert diverged actions in the male reproductive system, because it has been reported to stimulate Leydig-cell development and steroidogenic protein expression while at the same time inhibiting spermatogenesis in rats. ^34^ Other studies have also shown that prolonged or higher exposure to piperine may disturb testicular hormones, antioxidant status, and germ-cell integrity, indicating that its reproductive actions may vary according to dose, duration, and the specific endpoint assessed. ^42^ Therefore, the more favorable behavioral response at the lower dose and the stronger endocrine/sperm-quality response at the higher dose in the present study may reflect different dose thresholds for neural–vascular versus testicular effects, rather than a simple linear dose-response relationship. This interpretation should also be viewed in light of the short duration of exposure and the absence of direct phytochemical profiling of the tested extract, both of which limit precise mechanistic explanation. Taken together, the present findings suggest that black pepper fruit extract may exert selective early benefits in diabetic male reproductive dysfunction, but the optimal dose may differ depending on whether the primary target is sexual behavior, erectile function, or sperm/testicular outcomes.

Several limitations of this study should be considered. The alloxan-induced model represents an insulin-deficient, type 1-like diabetic state, and the 9-day experimental period reflects only short-term changes rather than established chronic diabetic sexual dysfunction. The behavioral assessment was confined to CL, ML, and MF, without systematic evaluation of intromission-related parameters, which limits the completeness of the sexual behavior analysis. Histological assessment was performed only in the testis, so the effects of black pepper extract on other organs commonly affected by diabetes, including the penis, pancreas, kidney, and liver, could not be determined. In addition, hormonal and mechanistic evaluation was limited to ITT concentration and eNOS mRNA expression, while other relevant endocrine and biochemical markers, such as LH, FSH, PDE5 activity, and oxidative stress-related assays (including SOD, catalase, or MDA), were not investigated. However, some outcomes also showed relatively wide within-group variability especially SC outcome with large SD, which may be related to biological heterogeneity and the small sample size of the study. Taken together, these limitations indicate that the present findings should be interpreted cautiously, and that further long-term studies with broader mechanistic and phytochemical evaluation are required.

## Conclusion

In conclusion, black pepper ( *Piper nigrum L.*) fruit extract showed dose-dependent and endpoint-specific effects in alloxan-induced diabetic rats. The lower dose was associated with more favorable changes in TPR and libido-related parameters, whereas the higher dose showed clearer effects on ITT and selected sperm parameters, particularly SC and SNM. Both extract doses were associated with higher LC than the untreated diabetic group, and eNOS mRNA expression was directionally higher in all treatment groups. These findings suggest that black pepper fruit extract may influence selected sexual and reproductive parameters in a short-term diabetic rat model. However, longer-term studies with broader mechanistic evaluation are required before firm conclusions can be drawn.

## Data Availability

Figshare: Black pepper (Piper nigrum L.) fruit extract ameliorates erectile dysfunction in alloxan-induced diabetic rats. Available at: https://doi.org/10.6084/m9.figshare.30456353. The project contains the following underlying data:
• Erectile function parameters values on each group • Libido parameters values on each group • Sperm analysis parameters values on each group • Testicular histology parameters values on each group Figshare: Black pepper (Piper nigrum L.) fruit extract ameliorates erectile dysfunction in alloxan-induced diabetic rats. Available at: https://doi.org/10.6084/m9.figshare.30456353. The project contains the following underlying data:
• qPCR eNOS values on the subjects • ARRIVE guidelines - Author checklist
