# Supplementary material for: Effects of Black Pepper (Piper nigrum L.) Fruit Extract on Sexual Function, Hormonal Parameters, Sperm Parameters, Testicular Histology, and eNOS Expression in Alloxan-Induced Diabetic Rats
Source: F1000Res. 2026 Apr 29;15:38. Originally published 2026 Jan 9. [Version 2] doi: 10.12688/f1000research.172637.2 (PMC13191311; doi:10.12688/f1000research.172637.2)
Supplement: Peer review history

# Reviewer report: Reviewer response for version 2

**DOI:** 10.5256/f1000research.198879.r480266

**Published:** 2026-05-18

**Authors:** Onyinyechi Ruth Nwagwe

**Affiliations:** 1 Federal University, Oye-Ekiti,, Oye-Ekiti,, Nigeria

I have gone through the revised version, the authors really adhered to the suggestions given to them, so in essence, the work is making a lot of sense now. 

 Again, the authors should check their grammatical expressions of each sentence they make.

Is the work clearly and accurately presented and does it cite the current literature?

Partly

If applicable, is the statistical analysis and its interpretation appropriate?

Yes

Are all the source data underlying the results available to ensure full reproducibility?

Partly

Is the study design appropriate and is the work technically sound?

Partly

Are the conclusions drawn adequately supported by the results?

Yes

Are sufficient details of methods and analysis provided to allow replication by others?

Yes

Reviewer Expertise:

NA

I confirm that I have read this submission and believe that I have an appropriate level of expertise to confirm that it is of an acceptable scientific standard.

# Reviewer report: Reviewer response for version 2

**DOI:** 10.5256/f1000research.198879.r480267

**Published:** 2026-05-12

**Authors:** Patrick Ebele Obi

**Affiliations:** 1 Enugu State University of Science and Technology, Enugu, Enugu, Nigeria

1. Fasting blood glucose levels of 500mg/dl and above are unrealistic. 

 2. Grammatical error: ''Fresh black pepper fruit is collected from a farmer...'' should be corrected to '' Fresh black pepper fruits were collected from a farmer....''

Is the work clearly and accurately presented and does it cite the current literature?

Yes

If applicable, is the statistical analysis and its interpretation appropriate?

I cannot comment. A qualified statistician is required.

Are all the source data underlying the results available to ensure full reproducibility?

Yes

Is the study design appropriate and is the work technically sound?

Partly

Are the conclusions drawn adequately supported by the results?

Yes

Are sufficient details of methods and analysis provided to allow replication by others?

Yes

Reviewer Expertise:

■Phytoevaluation                                                                         ■ Phytochemistry                                                                     ■ Phytomedicine                                                                         ■ Drug Discovery and Development

I confirm that I have read this submission and believe that I have an appropriate level of expertise to confirm that it is of an acceptable scientific standard.

# Reviewer report: Reviewer response for version 1

**DOI:** 10.5256/f1000research.190377.r458225

**Published:** 2026-03-19

**Authors:** Oluwapelumi Micheal Ajiboye

**Affiliations:** 1 Babcock University, Ogun State, Nigeria

**Summary of the Article**

 This study investigates the potential therapeutic effects of *Piper nigrum* (black pepper) fruit extract on erectile dysfunction (ED) and associated reproductive parameters in alloxan-induced diabetic male rats. The experimental design includes five groups: normal control, diabetic control, extract-treated diabetic group, and a sildenafil-treated group as a positive control. Outcome measures include erectile function indices (mounting frequency, intromission frequency, total penile reflexes), libido parameters, sperm characteristics, and histological evaluation of testicular tissue.

 The authors report that the extract significantly improved certain erectile and libido parameters, particularly total penile reflexes, while effects on sperm indices were largely non-significant. Histological observations are interpreted as showing restoration of testicular architecture.

 While the topic is relevant and potentially publishable, the manuscript exhibits substantial methodological, statistical, interpretational, and editorial deficiencies. Significant revision is required before the work can meet publication standards.

**MAJOR COMMENTS**

 1. The manuscript states that data are non-parametric, yet both Kruskal–Wallis test and ANOVA are reported. This represents a fundamental methodological contradiction. (ANOVA + Tukey post hoc (if parametric), OR Kruskal–Wallis + Dunn’s test (if non-parametric))

 2. Several results are described as “increased” or “improved” despite p > 0.05. E.g. sperm parameters (p = 0.877) are interpreted as improvements.

 3. The term “gland penis” is incorrect. Should be "glans penis".

 4. The term "male kisses" is non-scientific. Rephrase appropriately.

 5. Include units for sperm count and fluid intake

 6. Avoid overgeneralization. 

 7. There is contradictory literature use, i.e. "piperine increases testosterone", and "piperine inhibits spermatogenesis". These two statements contradict each other.

 8. "ddp-4" should be written as "DDP-4"

 9. Establish the relevance of the oxidative stress pathway and nitric oxide signalling in your discussion.

 10. Conclusion is overstated. Conclude using the specific parameters evaluated. Correct typo such as "randmoized" to "randomized"

 11. The phrase "known to be the cause of....." used in the second paragraph of the discussion section should be rephrased as "....is associated with..... "

 12. The discussion attributes effects to increased testosterone and antioxidant activity. However, no biochemical assays (e.g., testosterone, SOD, catalase, MDA) were performed. Remove or clearly qualify speculative statements and limit discussion to observed data unless supported by references.

 13. Extremely large standard deviations (e.g., sperm count: ±52.9), which may indicate poor experimental control or insufficient sample size.

 14. The title was duplicated in reference 3. The author's name(s) are not included in reference 20, and two journal names were mentioned within the same reference. Some references have the journal names abbreviated, while some do not (be consistent).

Is the work clearly and accurately presented and does it cite the current literature?

Partly

If applicable, is the statistical analysis and its interpretation appropriate?

No

Are all the source data underlying the results available to ensure full reproducibility?

No source data required

Is the study design appropriate and is the work technically sound?

Partly

Are the conclusions drawn adequately supported by the results?

Partly

Are sufficient details of methods and analysis provided to allow replication by others?

Partly

Reviewer Expertise:

Phytomedicine; Functional Foods and Nutraceuticals; Endocrine Disorder (Diabetes mellitus and its complications); Neurodegeneration.

I confirm that I have read this submission and believe that I have an appropriate level of expertise to state that I do not consider it to be of an acceptable scientific standard, for reasons outlined above.

# Response

**Published:** 2026-04-14

**Authors:** Exsa Hardibrata

**Affiliations:** Surgery, Universitas Lampung Fakultas Kedokteran, Bandar Lampung, Lampung, Indonesia

**1. Comment: **The manuscript states that data are non-parametric, yet both Kruskal-Wallis test and ANOVA are reported. This represents a fundamental methodological contradiction. (ANOVA + Tukey post hoc (if parametric), OR Kruskal–Wallis + Dunn’s test (if non-parametric))

**Response:** We sincerely thank the reviewer for identifying this inconsistency. We agree that the original statistical description contained a methodological contradiction. Although the manuscript incorrectly stated that one-way ANOVA with LSD post hoc testing was used, the analyses actually performed in this study were Kruskal-Wallis tests followed by Dunn’s multiple-comparison post hoc tests, consistent with the non-parametric nature of the data. This error has now been corrected in the revised manuscript, and the statistical methods have been rewritten to accurately reflect the analyses conducted. We have also carefully reviewed the Results section and tables to ensure consistency with the corrected statistical approach.

**2. Comment: **Several results are described as “increased” or “improved” despite p > 0.05. E.g. sperm parameters (p = 0.877) are interpreted as improvements.

**Response:** We sincerely thank the reviewer for this careful and important comment. We agree that it is not appropriate to interpret non-significant differences as definite improvements. In response, we have revised the Results and Discussion sections to ensure that only statistically significant findings are described as significant improvements, whereas outcomes with p > 0.05 are now reported more cautiously as numerical differences without statistical significance. For example, the statements regarding sperm parameters have been corrected accordingly. We have also rechecked the manuscript throughout to ensure consistency between the statistical results and their interpretation.

**3. Comment: **The term “gland penis” is incorrect. Should be "glans penis".

**Response:** We sincerely thank the reviewer for this valuable comment. We agree that the correct anatomical term is “glans penis” rather than “gland penis.” The manuscript has been revised accordingly in the Materials and Methods section to ensure anatomical accuracy.

**4. Comment: **The term "male kisses" is non-scientific. Rephrase appropriately.

**Response:** We sincerely thank the reviewer for this valuable comment. We agree that the expression “male kisses” is not appropriate for scientific reporting of animal sexual behavior. In the revised manuscript, we have replaced this phrase with more formal and objective terminology to describe the observed behavior in the Libido Assessment subsection. We changed the sentence into “Courtship latency was defined as the time from opening of the partition until the male initiated investigatory or courtship behavior toward the female, including anogenital sniffing or contact with the female’s body.”

**5. Comment: **Include units for sperm count and fluid intake.

**Response: **We sincerely thank the reviewer for this valuable observation. We agree that the omission of units for sperm count and fluid intake reduced the clarity of data presentation. In response, we have revised the manuscript into units of millions/mL to include the appropriate units for these variables throughout the text and tables.

**6. Comment:** Avoid overgeneralization.

**Response**: We sincerely thank the reviewer for this valuable observation. We agree that the original manuscript contained several statements that were overly broad in relation to the data presented. In response, we have revised the manuscript throughout to ensure that the conclusions are stated more cautiously and are fully supported by the results. Specifically, we avoided definitive claims, reduced overstatement of non-significant findings, and clarified that the observed effects should be interpreted as dose-dependent, endpoint-specific, and preliminary findings in a short-term alloxan-induced diabetic rat model.

**7. Comment:** There is contradictory literature use, i.e. "piperine increases testosterone", and "piperine inhibits spermatogenesis". These two statements contradict each other.

**Response**: We sincerely thank the reviewer for this valuable comment. We agree that the earlier wording may have oversimplified the published evidence. In the revised manuscript, we clarified that the available literature suggests complex and context-dependent effects of piperine, rather than uniformly beneficial or uniformly harmful reproductive effects. We now explicitly state that piperine may enhance Leydig-cell function and steroidogenic activity while, under different experimental conditions, adversely affecting spermatogenesis. We therefore revised the Discussion to reflect that these findings represent different reproductive endpoints and may vary according to dose, exposure duration, and study conditions.

**8. Comment**: "ddp-4" should be written as "DDP-4"

**Response:** We sincerely thank the reviewer for this careful observation. We agree that the abbreviation should be written consistently in uppercase. In the revised manuscript, “dpp-4” has been corrected to “DPP-4” throughout the text.

**9. Comment**: Establish the relevance of the oxidative stress pathway and nitric oxide signalling in your discussion.

**Response: **We sincerely thank the reviewer for this important comment. In response, we further revised the Discussion to more explicitly establish the relevance of the oxidative stress pathway and nitric oxide signaling to the findings of the present study. Specifically, we clarified that hyperglycemia-induced oxidative stress can reduce nitric oxide bioavailability and impair eNOS-mediated endothelial signaling in the corpus cavernosum, which contributes to diabetic erectile dysfunction. We also strengthened the discussion linking the directional increase in eNOS mRNA expression after black pepper treatment with the possibility of partial restoration of endothelial NO signaling. In addition, we added relevant recent literature to support the mechanistic relationship among oxidative stress, endothelial dysfunction, and impaired erectile function in diabetes.

****

**10. Comment: **Conclusion is overstated. Conclude using the specific parameters evaluated. Correct typo such as "randmoized" to "randomized"

**Response:** We sincerely thank the reviewer for this valuable observation. We agree that the previous Conclusion was somewhat overstated. In the revised manuscript, we have rewritten the Conclusion to refer specifically to the measured outcomes, including total penile reflexes, libido-related parameters, intratesticular testosterone, sperm concentration, sperm morphology, Leydig cell count, and eNOS expression, rather than making broad claims about diabetic male reproductive dysfunction overall. We have also revised the language to ensure that the findings are interpreted as dose-dependent, endpoint-specific, and preliminary. However, we don’t used the term “randomized” in our manuscript anymore.

**11. Comment:** The phrase "known to be the cause of....." used in the second paragraph of the discussion section should be rephrased as "....is associated with..... "

**Response:** We sincerely thank the reviewer for this valuable comment. We agree that the original phrasing implied a stronger causal interpretation than was warranted. In the revised Discussion, we have replaced “known to be the cause of” with newer sentences in our revised manuscript.

**12. Comment: **The discussion attributes effects to increased testosterone and antioxidant activity. However, no biochemical assays (e.g., testosterone, SOD, catalase, MDA) were performed. Remove or clearly qualify speculative statements and limit discussion to observed data unless supported by references.

**Response: **We sincerely thank the reviewer for this valuable observation. We agree that the original Discussion contained mechanistic statements that could be interpreted as stronger than warranted by the available data. In the revised manuscript, we have clarified that intratesticular testosterone was directly measured and may therefore be discussed as an observed endpoint, whereas oxidative stress-related mechanisms were not directly evaluated, as no assays such as SOD, catalase, or MDA were performed. We therefore revised the Discussion to remove over-speculative language, qualify mechanistic interpretations more carefully, and restrict causal inferences to the measured outcomes. Where oxidative stress or antioxidant effects are mentioned, these are now presented explicitly as literature-supported hypotheses rather than direct findings of the present study. We have also included this into our limitation.

**13. Comment:** Extremely large standard deviations (e.g., sperm count: ±52.9), which may indicate poor experimental control or insufficient sample size.

**Response:** We sincerely thank the reviewer for this valuable comment. We acknowledge that some outcomes, particularly sperm count, showed relatively large standard deviations. We carefully re-examined the raw data and confirmed that these values were accurate and not due to transcription error. We elected to retain the original values to preserve transparency and to avoid underreporting biological variability. We agree that the relatively small group size may have contributed to the wide dispersion observed. Accordingly, we used non-parametric statistical analysis (Kruskal-Wallis followed by Dunn’s post hoc test) and revised the text to ensure that the findings are interpreted cautiously. However, we also include this topic into our limitation to keep our study transparent.

**14. Comment:** The title was duplicated in reference 3. The author's name(s) are not included in reference 20, and two journal names were mentioned within the same reference. Some references have the journal names abbreviated, while some do not (be consistent). Is the work clearly and accurately presented and does it cite the current literature?

**Response:** We sincerely thank the reviewer for this important comment. We have carefully re-examined the entire reference list and corrected the identified errors, including the duplicated title in Reference 3, the incomplete author and journal information in Reference 20, and inconsistencies in journal-name formatting. The references have now been standardized throughout the manuscript to ensure consistency and accuracy. Furthermore, we revised the Discussion extensively to strengthen the scientific presentation of the work and to ensure that the findings are interpreted in light of relevant and current literature.

# Reviewer report: Reviewer response for version 1

**DOI:** 10.5256/f1000research.190377.r458227

**Published:** 2026-03-13

**Authors:** Patrick Ebele Obi

**Affiliations:** 1 Enugu State University of Science and Technology, Enugu, Enugu, Nigeria

1. The title of the research article should not be too conclusive. "effects of black pepper fruit extract on erectile dysfunction in alloxan-induced .......'' should be more appropriate.

 2. Introduction: The authors did not write enough information about erectile dysfunction under the introduction.  A little more should be written.

 3. The preparation of experimental animal across groups is not consistent or not clearly stated. From the manuscript, black pepper extract was administered daily for eight days while the Sildenafil Citrate was administered only on the ninth day (ie one hour before observation of erectile function and libido assessment).

 4. There are some grammatical errors under '' Plant preparation" that need correction. For instance, "The fresh fruit **is** washed and rinsed until clean and then dried"

 5. The authors should include the fasting blood sugar levels of the animals before and after inducing diabetes. 

 6. The duration of the research (9 days) is too short giving that diabetes complications such as erectile dysfunction are not instantaneous but gradual. 

 7. Report on phytoconstituents of the black pepper extract is completely missing. Knowledge of the phytoconstituents will be needed.

 8. The authors initially stated under 'animal preparation' that the study used 30 male Sprague Dawley rats but later mentioned female rats under 'libido assessment'. I understand that erectile dysfunction is male related complication but all animal used for this study should be clearly listed.

 9. The research results were poorly discussed.

Is the work clearly and accurately presented and does it cite the current literature?

Yes

If applicable, is the statistical analysis and its interpretation appropriate?

I cannot comment. A qualified statistician is required.

Are all the source data underlying the results available to ensure full reproducibility?

Yes

Is the study design appropriate and is the work technically sound?

Partly

Are the conclusions drawn adequately supported by the results?

Yes

Are sufficient details of methods and analysis provided to allow replication by others?

Yes

Reviewer Expertise:

■Phytoevaluation                                                                         ■ Phytochemistry                                                                     ■ Phytomedicine                                                                         ■ Drug Discovery and Development

I confirm that I have read this submission and believe that I have an appropriate level of expertise to confirm that it is of an acceptable scientific standard, however I have significant reservations, as outlined above.

# Response

**Published:** 2026-04-14

**Authors:** Exsa Hardibrata

**Affiliations:** Surgery, Universitas Lampung Fakultas Kedokteran, Bandar Lampung, Lampung, Indonesia

**1. Comment: **The title of the research article should not be too conclusive. “effects of black pepper fruit extract on erectile dysfunction in alloxan-induced .......” should be more appropriate.

**Response: **We sincerely thank the reviewer for this valuable suggestion. We agree that the original title was overly conclusive in its wording. In response, we revised the title from “Black pepper (Piper nigrum L.) fruit extract ameliorates erectile dysfunction in alloxan-induced diabetic rats” to “Effects of Black Pepper (Piper nigrum L.) Fruit Extract on Sexual Function, Hormonal Parameters, Sperm Parameters, Testicular Histology, and eNOS Expression in Alloxan-Induced Diabetic Rats.” We believe that the revised title is more neutral, scientifically appropriate, and more representative of the full range of outcomes assessed in the present study.

**2. Comment: **Introduction: The authors did not write enough information about erectile dysfunction under the introduction. A little more should be written.

**Response:** We are grateful to the reviewer for this constructive comment. In response, we expanded the Introduction by adding further information on erectile dysfunction, particularly in the context of diabetes mellitus. The revised text now includes additional discussion of the pathophysiological mechanisms underlying diabetic erectile dysfunction, including endothelial dysfunction, oxidative stress, neuropathic changes, hormonal disturbances, and structural alterations in penile tissue. We believe that these additions provide a clearer clinical and biological rationale for the present study.

**3. Comment:** The preparation of experimental animal across groups is not consistent or not clearly stated. From the manuscript, black pepper extract was administered daily for eight days while the Sildenafil Citrate was administered only on the ninth day (ie one hour before observation of erectile function and libido assessment).

**Response:** We thank the reviewer for this valuable comment. We acknowledge that the treatment regimens were not described with sufficient clarity in the original manuscript. In the revised manuscript, we clarified that black pepper extract was administered daily for eight consecutive days as the test intervention, whereas sildenafil citrate was given as a single dose on the ninth day, one hour before erectile function and libido assessment, as an acute positive control. Thus, the difference in administration schedule was intentional and reflected the distinct role of sildenafil as a reference treatment for erectile function rather than as a chronic intervention comparable to black pepper extract. The Materials and Methods section, particularly the Animal Preparation subsection, has been revised accordingly for clarity.

**4. Comment**: There are some grammatical errors under “Plant preparation” that need correction. For instance, “The fresh fruit is washed and rinsed until clean and then dried”. **Response**: We thank the reviewer for this valuable comment. We carefully revised the Plant Preparation subsection to correct the grammatical errors and improve sentence structure, tense consistency, and overall clarity. In particular, the sentence noted by the reviewer has been revised to conform to formal scientific writing style.

**5. Comment:** The authors should include the fasting blood sugar levels of the animals before and after inducing diabetes.

**Response:** We sincerely thank the reviewer for this helpful suggestion. In response, we added the fasting blood glucose levels of the animals before and after alloxan induction to the revised manuscript. These data are presented as Table 1 in the Materials and Methods section under the Animal Preparation subsection and include both individual values and mean ± SD for each group. We believe that this tabular presentation provides a clear and detailed description of the glycemic changes and confirms successful induction of hyperglycemia in the experimental animals.

**6. Comment: **The duration of the research (9 days) is too short giving that diabetes complications such as erectile dysfunction are not instantaneous but gradual.

**Response:** We sincerely thank the reviewer for this valuable comment. We agree that the duration of the present study was relatively short and may not fully reflect the chronic progression of diabetes-related erectile dysfunction, which typically develops gradually over time. However, the selection of day 9 after alloxan induction as the evaluation time point was based on considerations related to the stability of the diabetic model. At this stage, the animals had already passed the period of acute systemic toxicity associated with alloxan administration, so the data obtained were intended to reflect a more stable sub-acute hyperglycemic state rather than direct drug-induced toxic effects. In addition, this time point was considered sufficient to allow early structural changes in pancreatic β-cells, stabilization of oxidative stress-related changes, and regulation of relevant gene expression, thereby providing a suitable basis for assessment of both pathological progression and the early effects of the test compound. To ensure consistency of the diabetic state before final outcome assessment, fasting blood glucose levels were confirmed on day 3 and day 7 after alloxan induction and used as inclusion criteria to verify that all study animals remained in a stable diabetic condition before parameter evaluation on day 9. Moreover, because one of the aims of this study was to evaluate gene expression, particularly eNOS mRNA expression, day 9 was considered an appropriate time point at which hyperglycemia-related oxidative stress and pro-inflammatory signaling pathways would already be active, while tissue injury was still in an early and potentially modifiable phase. This timing was also considered sufficient to allow the test compounds to interact with relevant biochemical and cellular pathways before more advanced and possibly irreversible structural damage developed. Nevertheless, we agree that this represents a short-term experimental study, and we have revised the manuscript to emphasize that the findings should not be interpreted as evidence of reversal of long-standing chronic diabetic complications. We have also acknowledged this issue as a limitation in the Discussion and indicated that future studies with longer treatment duration and follow-up are necessary.

**7. Comment:** Report on phytoconstituents of the black pepper extract is completely missing. Knowledge of the phytoconstituents will be needed.

**Response:** We sincerely thank the reviewer for this valuable comment. We agree that reporting the phytoconstituents of the black pepper extract is important to support interpretation of its biological activity. In response, we have now included LC-MS analysis of the extract used in this study. The corresponding analytical procedure has been described in the Materials and Methods section, and the results are presented in Table. These additions provide direct characterization of the tested extract and strengthen the revised manuscript.

**8. Comment:** The authors initially stated under “animal preparation” that the study used 30 male Sprague Dawley rats but later mentioned female rats under “libido assessment”. I understand that erectile dysfunction is male related complication but all animal used for this study should be clearly listed.

**Response: **We thank the reviewer for this comment. In the original Methods section, under the Animal Preparation subsection, we stated that the experimental animals were 30 male Sprague Dawley rats. However, we recognize that the later description of the mating test, which involved female rats as stimulus animals, may have caused confusion. Therefore, we revised the Methods section to clarify that all experimental and treated animals were male rats, whereas female rats were used only as stimulus animals during the libido assessment.

**9. Comment: **The research results were poorly discussed. Is the work clearly and accurately presented and does it cite the current literature?

**Response:** We sincerely thank the reviewer for this valuable comment. In response, we substantially revised the Discussion section to improve the clarity, depth, and accuracy of interpretation of the study findings. Specifically, we expanded the discussion of each major outcome, aligned the interpretation more closely with the data presented in the tables, and incorporated more current and relevant literature to support comparison with previous studies and to explain the possible biological mechanisms involved. We also revised several statements to ensure that the conclusions are presented more cautiously and accurately, particularly in view of the short-term nature of the diabetic model and the endpoint-specific effects observed. We believe that these revisions have improved the scientific presentation of the work and strengthened the overall clarity of the manuscript.

# Reviewer report: Reviewer response for version 1

**DOI:** 10.5256/f1000research.190377.r455996

**Published:** 2026-03-12

**Authors:** Onyinyechi Ruth Nwagwe

**Affiliations:** 1 Federal University, Oye-Ekiti,, Oye-Ekiti,, Nigeria

The Fig 1 did not  capture  the year 2025 and the peak number of people having Diabetes, so it must be stated. There was no significant evidence presented in the authors background section to justify pepperine in an animal model.So more referenced evidence to explain why the treatment is better. And it should come from invitro/invivo works.

 I didn't see the anaesthesia used for the rats after the treatment . at the Methods area, Kindly recast the first sentence. Specify the kind of rats u used whether male/female.

 Provide the full name of the individual who undertook identification of the plant material. Was a Voucher specimen deposited in a publicly available Hebarium?A deposition number should be included and full name of the hebarium.

 Which type of DMwas identified in the rats 24 hours after the alloxan induction? 8days treatment could it lower blood glucose and improve sexual function? Could it be too good to believe?

 Why was there no graph of the rats blood glucose levels?

 For the sexual behavioral study, Why didn’t you check for the intromission number and Latency. Please let the video recording be shown. The Histological aspect, Why did you work only on the Testis? What of other organs wouldn’t you have checked them since your work is  on Diabetes  to know the effect of Pepperine on them? Again , why was other sexual hormones measured apart from Testosterone.

 Some Biochemical parameters was not assessed like PDE5 activity, SOME ENZYMATIC ASSAYS ON Diabetes.

 Also, why did you not carry out the  bioactive composition of the plant?

 Check your tables well to avoid mistakes. Example, Sperm Morphology, Group 1: 75 should have a decimal point.

Is the work clearly and accurately presented and does it cite the current literature?

Partly

If applicable, is the statistical analysis and its interpretation appropriate?

Yes

Are all the source data underlying the results available to ensure full reproducibility?

Partly

Is the study design appropriate and is the work technically sound?

Partly

Are the conclusions drawn adequately supported by the results?

Yes

Are sufficient details of methods and analysis provided to allow replication by others?

Yes

Reviewer Expertise:

NA

I confirm that I have read this submission and believe that I have an appropriate level of expertise to confirm that it is of an acceptable scientific standard, however I have significant reservations, as outlined above.

# Response

**Published:** 2026-04-14

**Authors:** Exsa Hardibrata

**Affiliations:** Surgery, Universitas Lampung Fakultas Kedokteran, Bandar Lampung, Lampung, Indonesia

**1. Comment:** The Fig 1 did not capture the year 2025 and the peak number of people having Diabetes, so it must be stated.

**Response:** We sincerely thank the reviewer for this valuable suggestion. In response, we revised the Introduction to incorporate the most recent epidemiological data on diabetes mellitus from the International Diabetes Federation (IDF) Diabetes Atlas, 11th edition, published in 2025. We clarified that the latest global estimate available in this report refers to 2024, as no separate worldwide estimate specifically for 2025 is currently provided. In addition, we included the projected peak global burden of diabetes reported in the same source, which estimates that the number of adults living with diabetes will increase to 853 million by 2050.

**2. Comment: **There was no significant evidence presented in the authors background section to justify pepperine in an animal model.So more referenced evidence to explain why the treatment is better. And it should come from invitro/invivo works.

**Response:** We are grateful to the reviewer for this important and constructive comment. In response, we substantially revised the Introduction by adding a dedicated paragraph to strengthen the scientific rationale for the use of black pepper in the present animal model. Specifically, we incorporated additional in vitro and in vivo evidence regarding piperine, the principal bioactive alkaloid of Piper nigrum, to support the biological plausibility of black pepper extract as a candidate intervention. We also clarified in the revised text that these preclinical studies were included to provide mechanistic and experimental justification for the intervention, rather than to imply definitive therapeutic superiority.

**3. Comment: **I didn't see the anaesthesia used for the rats after the treatment. at the Methods area, Kindly recast the first sentence.

**Response:** We thank the reviewer for this valuable comment. We apologize that the procedure was not clearly described in the original manuscript. In the revised Methods section, we clarified the terminal procedure used after completion of the treatment period and all functional assessments. Specifically, the text has been revised to clearly state that the animals were euthanized in accordance with the approved institutional ethics protocol.

**4. Comment:** Specify the kind of rats u used whether male/female.

**Response:** We thank the reviewer for this comment. In the original Methods section, under the Animal Preparation subsection, we stated that the experimental animals were 30 male Sprague Dawley rats. However, we recognize that the later description of the mating test, which involved female rats as stimulus animals, may have caused some confusion. Therefore, we revised the Methods section to clarify that all experimental and treated animals were male rats, whereas female rats were used only as stimulus animals during the libido assessment.

**5. Comment**: Provide the full name of the individual who undertook identification of the plant material. Was a Voucher specimen deposited in a publicly available Hebarium?A deposition number should be included and full name of the hebarium.

**Response: **We thank the reviewer for this valuable comment. We agree that inclusion of the full identifier name, herbarium deposition details, and voucher specimen number would improve the botanical documentation of the study. However, these records were not available for the present work. We therefore clarified the available plant source information in the revised manuscript and acknowledged the absence of formal voucher documentation as a limitation of the study.

**6. Comment: **Which type of DM was identified in the rats 24 hours after the alloxan induction? 8 days treatment could it lower blood glucose and improve sexual function? Could it be too good to believe?

**Response:** We sincerely thank the reviewer for this important and thoughtful comment. We clarified in the revised manuscript that the alloxan-induced diabetic rat model used in this study represents an insulin-deficient, type 1-like diabetes model, because alloxan selectively damages pancreatic β-cells and is widely used to induce experimental insulin-deficient diabetes in rodents. We also agree that an 8-day treatment period is relatively short and may not fully reproduce the chronic pathophysiology of long-standing diabetes-related sexual dysfunction. Therefore, we revised the manuscript to emphasize that the present work should be interpreted as a short-term experimental study designed to evaluate early changes in hyperglycemia and sexual/reproductive parameters after alloxan induction, rather than as evidence of complete reversal of established chronic diabetic complications. In addition, we added a more cautious statement in the Discussion noting that, although early improvements in blood glucose and functional parameters may be observed in experimental animal studies, these findings should be interpreted carefully and require confirmation in studies with longer treatment duration and follow-up.

**7. Comment:** Why was there no graph of the rats blood glucose levels?

**Response:** We sincerely thank the reviewer for this helpful suggestion. In response, we added the fasting blood glucose levels of the animals before and after alloxan induction to the revised manuscript. These data are presented as Table 1, which includes both individual values and mean ± SD for each group. We believe that this tabular presentation provides a clear and detailed description of the glycemic changes and confirms successful induction of hyperglycemia in the experimental animals.

**8. Comment: **For the sexual behavioral study, Why didn’t you check for the intromission number and Latency. Please let the video recording be shown.

**Response:** We sincerely thank the reviewer for this valuable comment. We agree that intromission number and intromission latency are relevant parameters in sexual behavior studies. In the present study, however, the behavioral assessment was prospectively focused on courtship latency, mount latency, and mount frequency as the predefined libido-related outcomes, and intromission-related parameters were therefore not systematically collected for quantitative analysis. We acknowledge this as a limitation and have clarified this point in the revised manuscript. In addition, the behavioral observations were performed using CCTV-based recording, and in response to the reviewer’s suggestion, we have provided representative recording examples and images in our Figshare (https://doi.org/10.6084/m9.figshare.30456353) as supporting documentation.

**9. Comment:** The Histological aspect, Why did you work only on the Testis? What of other organs wouldn’t you have checked them since your work is on Diabetes to know the effect of Pepperine on them?

**Response:** We sincerely thank the reviewer for this valuable comment. The histological evaluation in the present study was intentionally limited to the testis, because the primary objective of the study was to assess the effects of black pepper extract on male sexual and reproductive function rather than on general diabetic organ injury. Since sperm parameters, testosterone levels, and sexual behavior were central outcomes of this study, testicular histology was considered the most relevant tissue-level endpoint. Nevertheless, we agree that examination of other organs commonly affected by diabetes, including the pancreas, kidney, liver, and penile tissue, would have strengthened the study by providing additional evidence on the systemic effects of the extract. We have therefore acknowledged this as a limitation in the revised manuscript.

**10. Comment:** Again, why was other sexual hormones measured apart from Testosterone. Some Biochemical parameters was not assessed like PDE5 activity, SOME ENZYMATIC ASSAYS ON Diabetes.

**Response: **We sincerely thank the reviewer for this valuable comment. We agree that inclusion of additional sexual hormones and biochemical parameters would have strengthened the study. In the present work, testosterone was chosen as the principal hormonal marker because it is closely related to libido, erectile function, spermatogenesis, and Leydig cell activity, which were central outcomes of this study. We acknowledge that additional hormones, such as LH and FSH, as well as biochemical markers related to erectile function and diabetes, including PDE5 activity and other relevant enzymatic assays, were not evaluated. This limitation has now been stated in the revised manuscript, and we indicated that future studies should investigate these parameters to provide a more comprehensive mechanistic understanding.

**11. Comment:** Also, why did you not carry out the bioactive composition of the plant? **Response:** We sincerely thank the reviewer for this valuable comment. We agree that analysis of the bioactive composition of the black pepper extract is important for strengthening the mechanistic interpretation of the study. In response, we have revised the manuscript to include LC-MS characterization of the extract used in this study. The LC-MS procedure is now described in the Materials and Methods section with results presented in table. These additions improve the phytochemical documentation of the tested extract and provide direct characterization of the plant material used in the experiment. 12. Comment: Check your tables well to avoid mistakes. Example, Sperm Morphology, Group 1: 75 should have a decimal point. Response: We sincerely thank the reviewer for this valuable comment. In response, we carefully re-examined all tables in the manuscript and corrected the identified errors. In particular, the value reported for Sperm Morphology in Group 1 has been revised to include the appropriate decimal point. We also performed an additional review of all tabulated results to minimize formatting and transcription errors in the revised version.
